# Spatial Heterogeneity and Drivers of Heavy Metals in Soils and Sediments of the Nyangqu River Basin, Tibetan Plateau, China: Insights from GeoDetector and Explainable Machine Learning

**DOI:** 10.3390/toxics14080697

**Published:** 2026-08-06

**Authors:** Jiale Chen, Geng Xu, Duo Bu, Xiaomei Cui, Junli Chen, Qiangying Zhang, Bo Fang

**Affiliations:** 1 Key Laboratory of Biodiversity and Environment on the Qinghai-Tibet Plateau, Ministry of Education, School of Ecology and Environment, Xizang University, Lhasa 850000, China; 18715060764@163.com (J.C.); phudor@vip.163.com (D.B.); cuixiaomei@utibet.edu.cn (X.C.); 2 Analysis and Testing Center, Xizang University, Lhasa 850000, China; xgydsa@163.com (G.X.); chenjunli@utibet.edu.cn (J.C.)

**Keywords:** Nyangqu River Basin, heavy metals, ecological risk, GeoDetector, explainable machine learning

## Abstract

Heavy-metal contamination in alpine agricultural watersheds reflects interacting geological, environmental, and anthropogenic controls. In this study, arsenic (As), copper (Cu), lead (Pb), zinc (Zn), and chromium (Cr) were investigated in 213 farmland soil and sediment samples collected from the Nyangqu River Basin during 2019–2021 and 2024–2025. Pollution status and ecological risks were evaluated using the Nemerow Integrated Pollution Index (*Pn*) and Håkanson Potential Ecological Risk Index (RI), while potential factors associated with spatial variation were explored using GeoDetector and an explainable machine-learning framework integrating XGBoost, SHAP, and LIME. Mean As, Cu, Zn, and Cr concentrations exceeded Tibetan soil background values, whereas mean Pb remained below background. Farmland soils exhibited higher concentrations of As, Pb, and Zn than sediments, whereas Cu displayed comparable levels between the two media. Among the investigated metals, As showed persistent enrichment, whereas Cr exhibited the greatest spatial variability and strongest local anomalies. Overall, slight pollution dominated the study area (69.0%; median *Pn* = 1.73), although several hotspots increased the mean *Pn* to 2.10, indicating moderate pollution at the regional scale. After applying coefficient-adjusted thresholds (23/133), the ecological risk index (RI) ranged from 16.45 to 54.24 (mean = 32.70), with 5.6%, 93.9%, and 0.5% of samples categorized as low, moderate, and considerable risk, respectively, and no samples exhibiting high risk. The associated environmental factors showed element-specific patterns, involving soil physicochemical conditions, geological background, and localized anthropogenic indicators. Notably, factor combinations generally showed greater explanatory power than individual covariates, suggesting stronger joint statistical associations with heavy-metal spatial differentiation.

## 1. Introduction

Heavy metals are among the most persistent and hazardous contaminants in terrestrial and aquatic environments because of their toxicity, non-degradability, and tendency to accumulate in environmental media. In watershed systems, soils and sediments serve as major reservoirs and transfer pathways of heavy metals, linking terrestrial inputs with aquatic transport and redistribution processes [1,2,3]. Once introduced into the environment, heavy metals can undergo complex biogeochemical transformations and accumulate across different media, posing long-term threats to agricultural productivity, ecosystem stability, and human health [4,5,6]. Consequently, understanding the occurrence, ecological risks, and spatial heterogeneity of heavy metals has become a critical issue for watershed environmental management.

Alpine agricultural watersheds present particular challenges for heavy-metal research because their spatial distributions are jointly influenced by natural geological backgrounds and increasing anthropogenic disturbances. Strong lithological controls, heterogeneous parent materials, and unique soil-forming processes often result in elevated natural background concentrations of heavy metals, whereas agricultural activities, irrigation practices, fertilizer application, and rural development may introduce additional anthropogenic inputs. Disentangling the relative contributions of these factors is therefore essential for understanding heavy-metal accumulation processes and identifying pollution hotspots in alpine environments. The Nyangqu River, a tributary of the Yarlung Zangbo River, drains one of the most important grain-producing areas on the Tibetan Plateau [7].

Previous studies have documented heavy-metal contamination characteristics in agricultural soils across this region. For example, Wang et al. [8] reported that farmland soils in this region generally exhibited good environmental quality, although Cd, Cu, and Pb showed clear anthropogenic influences. Zhu et al. [9] found that farmland soils in the region exhibited higher pollution levels and ecological risks than those in forestland, likely due to stronger human disturbances. Other studies have further assessed heavy-metal concentrations, spatial distributions, sources, and ecological risks in surface soils from the region [10,11,12], revealing enrichment trends in the Nyangqu River Basin and suggesting that agricultural activities may contribute to elevated ecological risks.

However, heavy-metal distributions in this alpine watershed cannot be explained solely by anthropogenic activities. Previous investigations have highlighted the strong influence of parent materials, lithological conditions, and regional geological settings on heavy-metal accumulation patterns [12,13], indicating that the basin retains substantial geogenic signatures. These findings suggest that the spatial heterogeneity of heavy metals in the Nyangqu River Basin results from complex interactions between natural background conditions and human activities. Nevertheless, the mechanisms governing such interactions remain insufficiently understood.

Several knowledge gaps still limit current understanding of heavy-metal behavior in alpine agricultural watersheds. First, most previous studies have focused on a single environmental medium, particularly farmland soils, while comparative evidence between farmland soils and river sediments remains limited. Given that sediments record pollutant transport, deposition, and remobilization processes, integrated analyses across multiple media are necessary to reveal watershed-scale accumulation patterns. Second, existing studies have largely emphasized contamination levels, source identification, and ecological risks, whereas the drivers of spatial heterogeneity have received comparatively less attention. Third, traditional receptor models such as principal component analysis (PCA) and positive matrix factorization (PMF) can identify potential sources and estimate source contributions, but their interpretations often rely heavily on expert judgment and provide limited insight into nonlinear responses, interaction effects, and local hotspot formation in complex environmental systems.

Recent advances in spatial analysis and explainable machine learning offer new opportunities to address these challenges. GeoDetector can quantify the explanatory power of environmental factors and identify interaction effects without assuming predefined linear relationships. Studies have shown that interactions between environmental variables often explain heavy-metal enrichment more effectively than individual factors alone [14]. Meanwhile, explainable machine-learning approaches, such as eXtreme Gradient Boosting (XGBoost) combined with SHapley Additive exPlanations (SHAP), can improve predictive performance while revealing complex nonlinear relationships between environmental variables and heavy-metal concentrations [15]. Furthermore, integrated frameworks combining GeoDetector and explainable machine learning have been successfully applied to identify spatial drivers and interaction mechanisms of heavy metals [16,17]. By coupling basin-scale driver identification with local-scale interpretation, these approaches provide a promising pathway for understanding spatial heterogeneity and hotspot formation processes.

Therefore, this study focused on farmland soils and river sediments in the Nyangqu River Basin to elucidate heavy-metal distribution patterns, pollution characteristics, and their spatial determinants. An integrated analytical framework was established by coupling GeoDetector with machine-learning approaches, including XGBoost, SHAP, and Local Interpretable Model-agnostic Explanations (LIME), to quantify the contributions and interpretability of environmental controls across multiple dimensions, including soil physicochemical conditions, geological background, anthropogenic influences, and environmental media. Specifically, this study aimed to address three questions: (1) How do heavy-metal concentrations and ecological risks differ between farmland soils and river sediments? (2) Which environmental factors dominate the spatial differentiation of heavy metals at the watershed scale? (3) How do natural background conditions and anthropogenic disturbances interact in shaping localized enrichment patterns? By integrating cross-media comparisons, watershed-scale driver identification, and interpretable hotspot analysis, this study provides new insights into the spatial heterogeneity of heavy metals in alpine agricultural watersheds and provides a scientific basis for targeted pollution mitigation and precision environmental management.

## 2. Materials and Methods

### 2.1. Study Area

The Nyangqu River Basin is located in the southern Tibetan Plateau (28°10′–29°20′ N and 88°35′–90°15′ E), covering Kangmar County, Gyantse County, Bailang County, and Samzhubzê District of Shigatse, Xizang, China. The basin covers approximately 11,130 km^2^, with elevations ranging from 3822 to 6967 m and a main channel length of approximately 217 km [18,19]. The terrain generally decreases from the southeastern headwaters to the middle and lower reaches, where the river valley widens and agri-cultural activities are concentrated [18].

The basin experiences a temperate semi-arid plateau monsoon climate, with a mean annual temperature of 4–6 °C and annual precipitation of 300–365 mm [18,19]. The geological setting is dominated by sedimentary strata of the Tethyan Himalaya, with locally distributed metamorphic and igneous rocks from adjacent tectonic units [20]. Variations in lithology, parent materials, and weathering processes contribute to spatial heterogeneity in soil and sediment geochemical backgrounds [13,20]. Seasonal precipitation also promotes runoff generation, soil erosion, and sediment transport, potentially affecting the redistribution of heavy metals within the basin [19,21]. The dominant soil types include semi-Luvisols, dark semi-hydromorphic soils, and hydromorphic soils [18]. Highland barley, wheat, and rapeseed are the major crops, highlighting the importance of the basin for agricultural production in Xizang [7,18].

### 2.2. Sample Collection, Pretreatment, and Heavy Metal Determination

**Figure 1:**
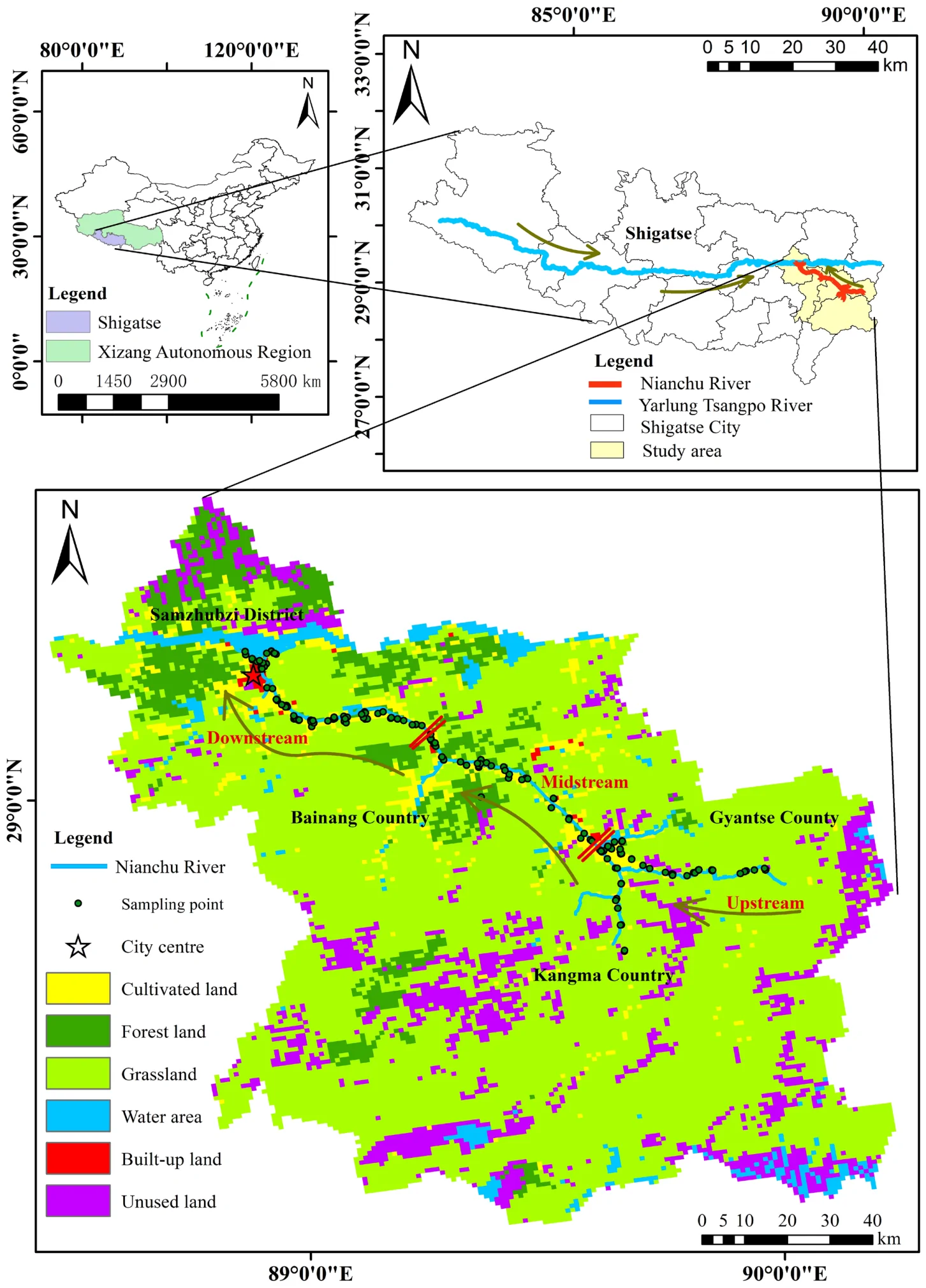
Study area and sampling-site distribution in the Nyangqu River Basin. The two pairs of red solid lines denote the boundaries between the upper, middle, and lower reaches, and the arrows indicate the flow direction of the Nianchu River.

A total of 213 samples were collected from the Nyangqu River Basin during 2019–2021 and 2024–2025, including 149 farmland soil samples and 64 river sediment samples. Farmland soil samples were collected following the Technical Specification for Soil Environmental Monitoring (HJ/T 166–2004) [22], and the geographic coordinates of each sampling site were recorded using a global positioning system (GPS). Specifically, surface soil samples (0–20 cm) were collected from the four corners and the center of each 10 m × 10 m quadrat and subsequently homogenized to obtain a composite sample. After quartering, approximately 1 kg of soil was sealed in a polyethylene bag and transported to the laboratory. Sediment samples were collected from depositional zones with relatively slow water flow or from representative river reaches, while areas affected by severe bed scouring, unstable sediment accumulation, or surface disturbance were avoided. Surface sediments (0–5 cm) were collected using a stainless-steel shovel. The locations of all sampling sites are presented in Figure 1.

All samples were air-dried at room temperature in the laboratory. Visible stones, plant debris, and other foreign materials were removed manually. The dried samples were then ground and passed through a 100-mesh nylon sieve prior to chemical analysis. Sample digestion and heavy metal determination were conducted according to HJ 1315-2023 [23]. Briefly, approximately 0.2 g of each dried and sieved sample was weighed into a microwave digestion vessel, followed by the addition of 9 mL HNO_3_, 3 mL HCl, 2 mL HF, and 1 mL HClO_4_. Digestion was conducted using a microwave digestion system (MARS 6 CLASSIC, CEM Corporation, Matthews, NC, USA), and the digestion program is detailed in Supplementary Table S1. After digestion, the solution was transferred to an acid-removal instrument (TK20, SINEO, Shanghai, China) and heated at 120 °C until near dryness. The residues were subsequently diluted to 50 mL with ultrapure water in polyethylene volumetric containers.

The concentrations of As, Cu, Pb, Zn, and Cr were determined using inductively coupled plasma mass spectrometry (ICP–MS; NexION 1000G, PerkinElmer, Shelton, CT, USA). Prior to instrumental analysis, all digested solutions were filtered through 0.22 μm membrane filters. Quality assurance and quality control (QA/QC) procedures were implemented throughout the analytical process. The certified reference material GBW07548 (GSS-57, Shigatse, Xizang, China) was analyzed together with the samples using the same digestion and analytical procedures. The analytical results for the certified reference material are presented in Supplementary Table S2. The measured concentrations of As, Cu, Pb, Zn, and Cr agreed well with the certified values, with recoveries ranging from 88.9% (Zn) to 111.7% (Cu). All samples were analyzed in triplicate, and the average values were used for subsequent analyses. Relative standard deviations (RSDs) were below 5% for all target elements, while recoveries of the ICP–MS internal standard (Rh) ranged from 80% to 120%, demonstrating satisfactory analytical precision and accuracy.

### 2.3. Selection and Spatial Quantification of Environmental Covariates

To identify the drivers of heavy-metal spatial differentiation in the Nyangqu River Basin and distinguish the influences of soil physicochemical conditions and anthropogenic activities, an integrated environmental covariate framework was established. The framework incorporated four categories of explanatory variables: soil physicochemical variables, geological background, anthropogenic influences, and environmental media. Soil physicochemical and geological variables represented regional pedogenic processes and parent-material heterogeneity, respectively, whereas anthropogenic variables captured the potential impacts of human activities, particularly transportation and socioeconomic development. Medium type was included to account for differences between agricultural soils and river sediments. Except for relatively stable soil physicochemical and geological attributes, all time-sensitive spatial datasets were matched to the corresponding sampling periods to ensure temporal consistency.

The soil physicochemical variables included pH, soil organic matter (SOM), total nitrogen (TN), total phosphorus (TP), total potassium (TK), alkali-hydrolysable nitrogen (AN), available phosphorus (AP), and available potassium (AK) were obtained from a published national-scale soil-property dataset with a 1 km spatial resolution and extracted according to the geographic coordinates of the sampling sites. To evaluate the potential for spatial pseudo-replication, we conducted an additional spatial matching assessment. The 213 sampling sites were assigned to 111 unique 1 km grid cells, with 49 grid cells containing multiple sampling sites (151 samples in total) and the remaining 62 grid cells containing only one sampling site each. Surface soil properties (0–4.5 cm) were used to represent near-surface soil conditions in subsequent spatial driver analyses. Geological background was characterized using the following parent material and lithological data, including Quaternary deposits (QDep), mixed Jurassic–Cretaceous geological units (JKMix), sedimentary rocks (Sed), and metamorphic–volcanic rocks (MetaVol). These categorical variables were converted into binary indicators (1 = presence, 0 = absence) and assigned to each sampling site. As geological characteristics are temporally stable, no temporal adjustment was applied. Anthropogenic influences were represented by road distance (Road_Dist), population density (Pop), and nighttime light intensity (NTL). Point-of-interest (POI) data were further incorporated to capture localized human activity intensity, including automobile repair (AutoRep), motorcycle repair (MotoRep), transportation services (Transport), catering services (Catering), daily-life services (LifeServ), shopping services (Shop), and enterprises (Firm). The spatial distribution of POI categories was quantified using kernel density estimation (KDE).

Considering sample size, model stability, and the need to capture basin-scale driving patterns, farmland soils and sediments were jointly analyzed. A media-type variable was incorporated to characterize environmental heterogeneity between the two media, with farmland soils coded as 1 and sediments coded as 0. This variable was included to represent media-specific effects within a pooled-sample framework, allowing the identification of overarching drivers at the basin scale. Detailed data sources and spatial resolutions for all environmental covariates are provided in Table 1.

### 2.4. Pollution and Potential Ecological Risk Assessment

The single-factor pollution index (*Pi*) and Nemerow Integrated Pollution Index (*Pn*) were used to evaluate the accumulation and overall pollution levels of multiple heavy metals in samples from the study area [24]. The detailed calculation procedures and classification criteria for the pollution indices (*Pi* and *Pn*) and ecological risk index (RI) are described in Supplementary Text S1. The RI was calculated according to the following equation [25]:

(1) $$RI=\sum_{i=1}^{n}E_{r}^{i}=\sum_{i=1}^{n}T_{r}^{i}\times \frac{C_{i}}{S_{i}}$$

where $T_{r}^{i}$ represents the toxic-response coefficient of heavy metal *i*. The toxic-response coefficients were assigned as As (10), Cu (5), Pb (5), Cr (2), and Zn (1). *C_i_* is the measured concentration of heavy metal *i* in the sample, and *S_i_* is the corresponding background or reference value. $E_{r}^{i}$ denotes the potential ecological risk index of heavy metal *i*, and RI represents the comprehensive potential ecological risk index for a given sampling site. Because the five metals analyzed in this study (As, Cu, Pb, Zn, and Cr) had a summed toxic-response coefficient of 23, compared with 133 in the original eight-substance Håkanson framework, the conventional RI thresholds (150, 300, and 600) were proportionally adjusted by a factor of 23/133 [26]. The adjusted classification thresholds are provided in Supplementary Text S1.

### 2.5. GeoDetector Analysis

GeoDetector is a statistical framework for quantifying spatial stratified heterogeneity and evaluating the explanatory power of environmental factors for spatial patterns of response variables [27,28]. It is based on the assumption that if an explanatory factor influences the spatial distribution of a response variable, the spatial stratification of the factor should correspond to reduced within-stratum variance of the response variable. Unlike conventional regression approaches, GeoDetector does not require predefined linear relationships, making it suitable for investigating complex and non-linear environmental processes [27,28]. In this study, GeoDetector analysis was performed using Python-based implementations and applied separately to As, Cu, Pb, Zn, and Cr. The factor detector was used to quantify the individual explanatory power of each environmental covariate, while the interaction detector was employed to evaluate whether the combined effects of two factors enhanced, weakened, or independently explained heavy-metal spatial variability.

Before GeoDetector analysis, continuous environmental variables were discretized using quantile-based classification with five classes; when this was not feasible, equal-interval classification with five classes was applied. The explanatory power of each factor was quantified using the *q*-statistic:

(2) $$q=1-\frac{\sum_{h=1}^{L}N_{h}\sigma_{h}^{2}}{N\sigma^{2}}=1-\frac{SSW}{SST}$$

(3) $$SSW=\sum_{h=1}^{L}N_{h}\sigma_{h}^{2},\;SST=N\sigma^{2}$$

where *q* denotes the explanatory power statistic, ranging from 0 to 1. Higher *q* values indicate greater explanatory power of the factor to the observed spatial heterogeneity. *L* represents the number of strata for variable or factor *X*, and *h* denotes a specific stratum classified according to the environmental factor. *N_h_* and *N* are the number of samples within stratum *h* and across the entire study area, respectively. $\sigma_{h}^{2}$ and *σ*^2^ denote the variance of heavy-metal concentrations within stratum *h* and across the whole study area, respectively. *SSW* and *SST* represent the within-stratum sum of squares and the total sum of squares, respectively.

For the interaction detector, the spatial strata of two explanatory factors (X_1_ and X_2_) were overlaid to generate a combined stratification (X_1_ ∩ X_2_), and the corresponding interaction *q*-statistic was calculated. The interaction effect was determined by comparing *q*(X_1_ ∩ X_2_) with *q*(X_1_), *q*(X_2_), and *q*(X_1_) + *q*(X_2_), thereby identifying whether the two factors exhibited independent effects, bi-factor enhancement, nonlinear enhancement, or weakening effects. The detailed classification criteria are provided in Supplementary Text S2 and Table S3.

### 2.6. XGBoost Modeling and Interpretation Methods

#### 2.6.1. Extreme Gradient Boosting (XGBoost)

XGBoost was employed to model nonlinear relationships between environmental covariates and heavy-metal concentrations and to evaluate predictor importance. This regularized gradient boosting framework can capture complex nonlinear interactions among variables while improving model generalization and reducing overfitting risk [29]. The model prediction function, objective function, and regularization term are expressed in Equations (4)–(6).

(4) $${\hat{y}}_{i}=\sum_{k=1}^{K}f_{k}(x_{i}),\;f_{k}\in F$$

(5) $$Obj=\sum_{i=1}^{n}l(y_{i},{\hat{y}}_{i})+\sum_{k=1}^{K}\Omega (f_{k})$$

(6) $$\Omega (f)=\gamma T+\frac{1}{2}\lambda \sum_{j=1}^{T}w_{j}^{2}$$

where *y_i_* and ${\hat{y}}_{i}$ represent the observed and predicted values of the *i*-th sample, respectively; *K* denotes the total number of decision trees; *f_k_* represents the function of the *k*-th tree; and *x_i_* is the feature vector of the *i*-th sample. *Obj* denotes the objective function to be minimized, *n* is the total number of training samples, and *l* is the loss function used to measure the difference between the observed and predicted values of the *i*-th sample. Ω(*f_k_*) represents the regularization term used to reduce overfitting. *γ* is the penalty coefficient for the number of leaf nodes, *T* denotes the number of leaves in a tree, *λ* is the L2 regularization coefficient, and *w_j_* represents the weight of the *j*-th leaf node.

To examine the sensitivity of model performance to data splitting strategies, three training/testing ratios (8:2, 7:3, and 6:4) were tested independently for each heavy metal. Hyperparameter tuning was performed using GridSearchCV [30] with K-fold cross-validation [31], as implemented in scikit-learn (version 1.5.2), on the training dataset. The optimal hyperparameter combination for each heavy-metal model was selected according to the cross-validation performance and subsequently used to construct the final predictive model. The optimized XGBoost models were subsequently interpreted using SHapley Additive exPlanations (SHAP) to quantify the relative importance of environmental covariates and characterize their directional associations with model predictions. Detailed model settings are provided in Supplementary Text S3.1 and Table S4.

#### 2.6.2. Evaluation Metrics

The coefficient of determination (*R*^2^) was used to quantify the proportion of variance explained by the model. A value closer to 1 indicates better model fitting [32]. The root mean square error (RMSE) summarizes the magnitude of prediction errors in the original measurement units, with lower values indicating smaller prediction errors; a smaller RMSE indicates higher prediction accuracy [33].

(7) $$R^{2}=1-\frac{\sum_{i=1}^{n}{\left(y_{i}-{\hat{y}}_{i}\right)}^{2}}{\sum_{i=1}^{n}{\left(y_{i}-\bar{y}\right)}^{2}}$$

(8) $$\mathrm{RMSE}=\sqrt{\frac{1}{n}\sum_{i=1}^{n}{\left(y_{i}-{\hat{y}}_{i}\right)}^{2}}$$

where *y_i_* is the observed value, ${\hat{y}}_{i}$ is the predicted value, *n* is the sample size, and $\bar{y}$ is the mean observed value.

#### 2.6.3. SHAP

To enhance the interpretability of XGBoost models and quantify the contribution of environmental covariates to model predictions, SHAP was applied. SHAP is a model-agnostic interpretation framework derived from Shapley values in cooperative game theory, which attributes model predictions to individual input features while ensuring local accuracy and consistency [34]. By decomposing model outputs into additive feature contributions, SHAP enables the relative importance of environmental covariates and reveals the direction and magnitude of their effects on model predictions. The SHAP explanatory model is defined as:

(9) $$f(x)=\phi_{0}+\sum_{j=1}^{m}\phi_{j}$$

where *f*(*x*) is the prediction of the explanatory model for sample *x*, *ϕ*_0_ is the model baseline value, *m* is the total number of input features, and *ϕ_j_* is the SHAP value of the *j*-th feature. A positive SHAP value indicates that the feature contributes to increasing the predicted value, whereas a negative SHAP value indicates that it contributes to decreasing the prediction.

#### 2.6.4. LIME

To further characterize local contribution patterns for representative high-concentration samples, LIME was used for local interpretation. Representative high-concentration samples were selected based on predefined concentration criteria. Detailed selection criteria and candidate sample numbers are provided in Supplementary Text S3.2 and Table S5. LIME constructs a locally interpretable surrogate model in the neighborhood of a target sample to approximate the local behavior of the original complex model in the local feature space [35]. Formally, the objective function is expressed as follows:

(10) $$\xi (x)=\arg\min_{g\in G}\left[L(f,g,\pi_{x})+\Omega (g)\right]$$

where *ξ*(*x*) is the local interpretable model generated for sample *x*; *G* is the family of all candidate interpretable models; *g* is a specific interpretable surrogate model within *G*; *f* is the original complex black-box model; *π_x_* is a weighting function specifying the local neighborhood of sample *x*; *L*(*f*, *g*, *π_x_*) is the loss function measuring the degree to which *g* approximates *f* within the local space defined by *π_x_*; Ω(*g*) is the complexity penalty that ensures the surrogate model remains sufficiently simple and interpretable.

### 2.7. Software and Computational Environment

The software and computational packages used for data processing, spatial analysis, statistical modeling, model interpretation, and visualization are summarized in Table 2.

## 3. Results and Discussion

### 3.1. Heavy-Metal Concentration Levels and Spatial Differentiation

**Figure 2:**
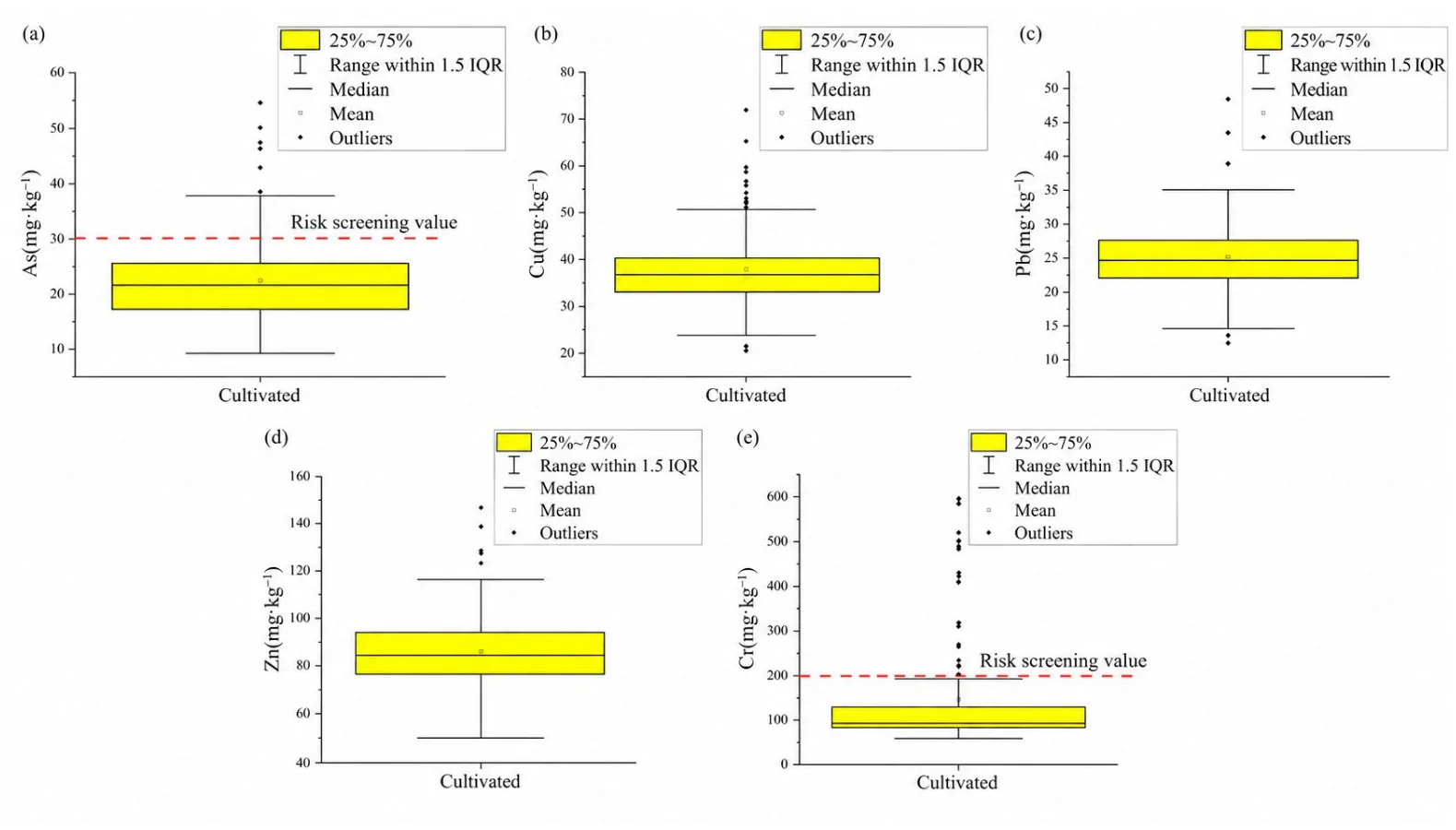
Boxplots of heavy metal concentrations in cultivated soils (**a**–**e**).

**Figure 3:**
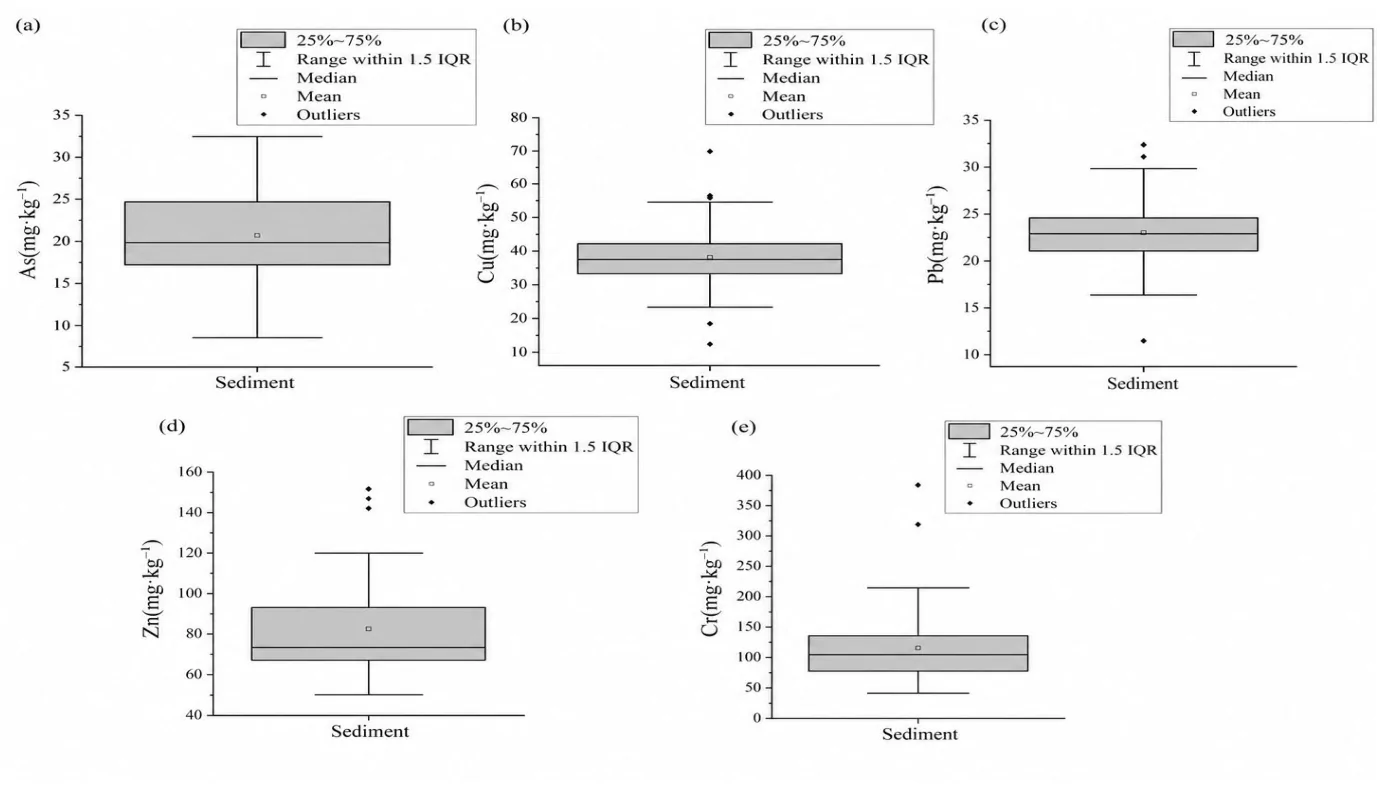
Boxplots of heavy metal concentrations in sediments (**a**–**e**).

In our previous investigation, eight heavy metals, including As, Cu, Pb, Zn, Cr, Ni, Cd, and Hg were analyzed. The results showed that Ni, Cd, and Hg were below detection limits in most samples and exhibited limited spatial variability. Furthermore, exploratory XGBoost models for these three elements showed no meaningful out-of-sample predictive ability, with a test-set *R*^2^ of −0.0008. Therefore, Ni, Cd, and Hg were not included in the further analysis and discussion in the present study. The heavy-metal concentrations in the Nyangqu River Basin are summarized in Table 3. Previous studies reported that the pH of surface soils (0–20 cm) in the Nyangqu River Basin region of Xizang ranges from 7.25 to 7.41 [36]. Accordingly, the risk-screening values specified for soils with 6.5 < pH ≤ 7.5 in GB 15618–2018 [37] were adopted as the evaluation criteria. As shown in Table 3 and Figure 2 and Figure 3, the mean concentrations of As, Cu, Zn, and Cr during 2019–2021 and 2024–2025 generally exceeded the corresponding soil background values for Xizang, whereas Pb concentrations were predominantly below the regional background level. This result indicates distinct element-specific accumulation characteristics across the basin. Notably, As exhibited consistently elevated concentrations throughout the study period, while Cr showed substantial variability and pronounced spatial heterogeneity. When evaluated against the cultivated-land risk-screening criteria, Cu, Pb, and Zn remained below the regulatory thresholds at all sampling sites. In contrast, exceedances were observed primarily for As and Cr, highlighting these two elements as the dominant contributors to potential soil environmental risk. Importantly, the relatively low exceedance frequencies suggest that contamination is characterized by localized hotspots rather than widespread regional pollution. A comparison between cultivated soils and contemporaneous sediment samples further revealed that the mean concentrations of As, Pb, and Zn were generally higher in soils than in sediments, whereas Cu exhibited only minor differences between the two media. Although Cr concentrations fluctuated considerably among sampling locations, several sites displayed exceptionally high values, indicating the presence of distinct local enrichment processes. Taken together, these findings demonstrate that As represents a persistent and basin-wide concern, while Cr is characterized by stronger spatial dispersion and site-specific anomalies. Therefore, As and Cr should be regarded as priority elements for monitoring and source verification, and receive particular attention in future monitoring, source identification, and risk management efforts.

#### Pollution and Ecological Risk Assessment

**Figure 4:**
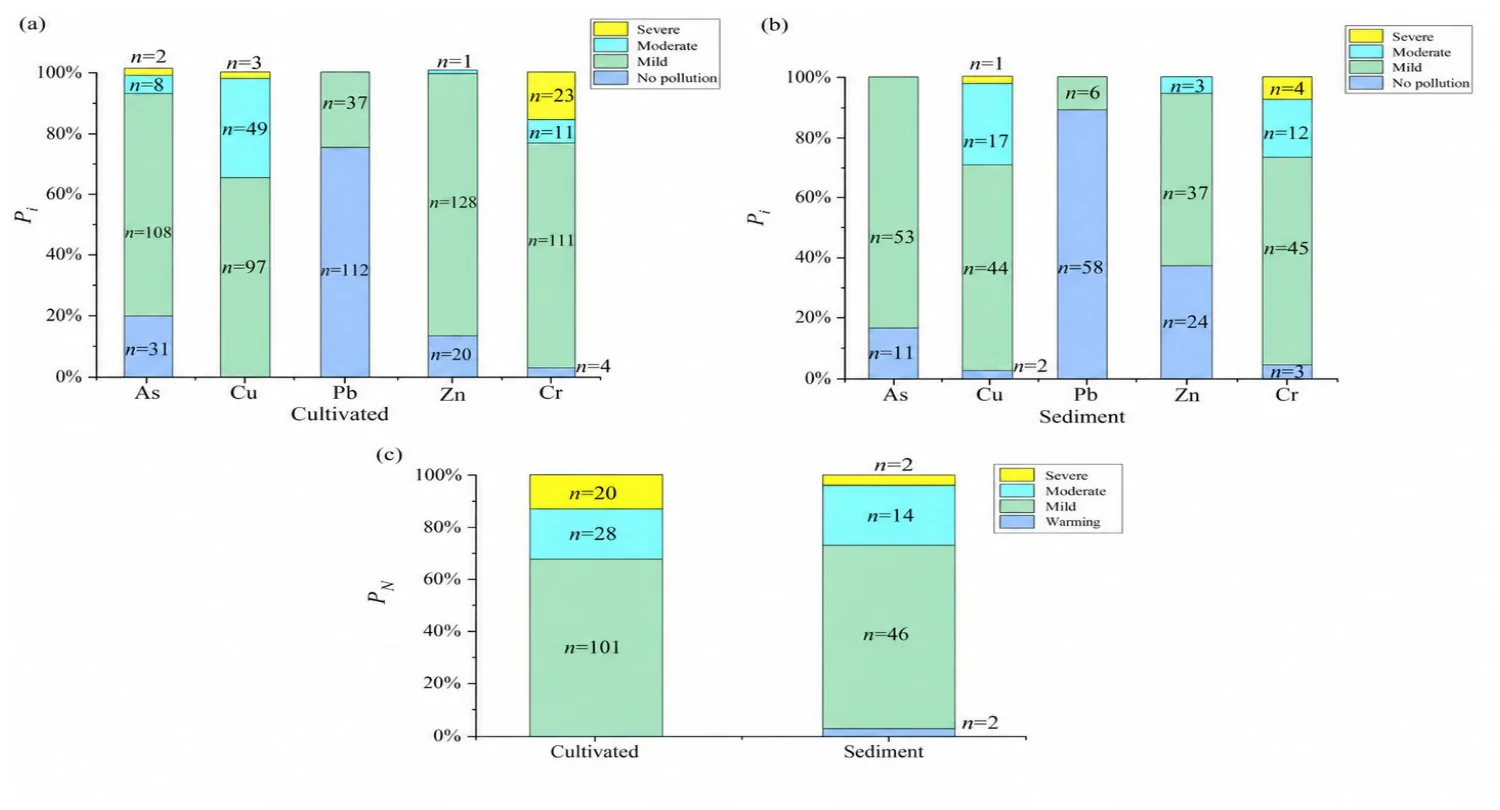
Pollution classification based on *Pi* and *Pn* across sampling media. (**a**) Distribution of single-factor pollution index (*Pi*) categories for As, Cu, Pb, Zn, and Cr in cultivated soils; (**b**) distribution of *Pi* categories for As, Cu, Pb, Zn, and Cr in sediments; (**c**) distribution of Nemerow Integrated Pollution Index (*Pn*) categories in cultivated soils and sediments. The values indicate the number of samples (*n*) in each pollution category.

The *Pn* values ranged from 0.86 to 6.49, with a mean of 2.10 and a median of 1.73. According to the classification criteria in Supplementary Text S1, the mean *Pn* indicated moderate pollution at the regional scale, whereas the median suggested that slight pollution represented the prevailing condition. Among individual sampling sites, 147 samples (69.0%) were categorized as slightly polluted, followed by moderate pollution (42 samples, 19.7%), heavy pollution (22 samples, 10.3%), and warning levels (2 samples, 0.9%). The predominance of slight pollution, together with the higher arithmetic mean than median, indicates that localized sites with moderate-to-heavy contamination exerted a disproportionate influence on overall pollution intensity. As illustrated in Figure 4, both cultivated soils and sediments were dominated by slight pollution. However, cultivated soils exhibited a higher proportion of moderate- and heavy-pollution sites than sediments, suggesting greater overall contamination pressure in agricultural soils.

**Figure 5:**
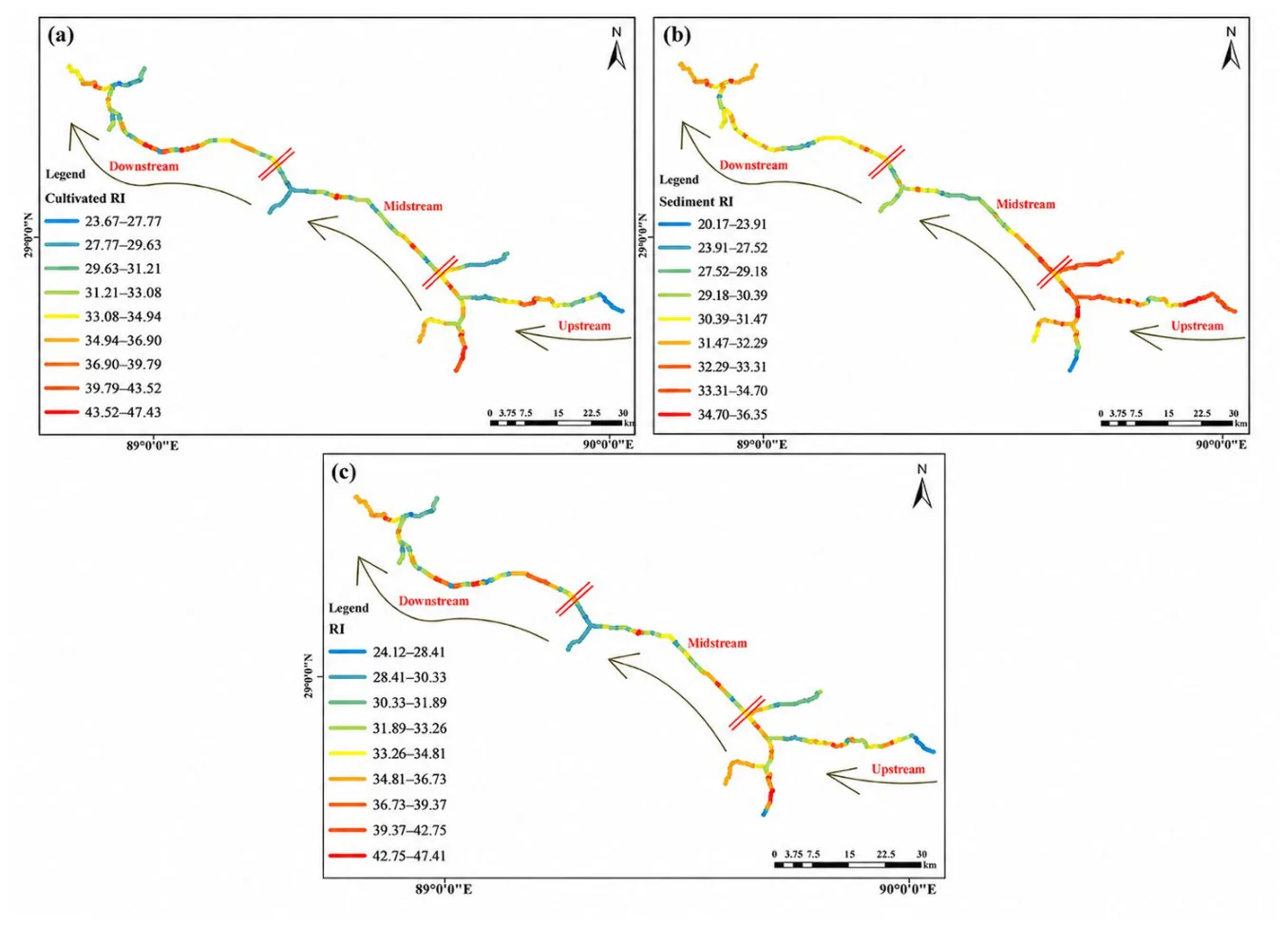
Spatial distribution of the potential ecological risk index (RI) across sampling media. (**a**) Spatial distribution of RI in cultivated soils; (**b**) spatial distribution of RI in sediments; (**c**) spatial distribution of RI for all samples, including cultivated soils and sediments.

The RI results indicated that potential ecological risk was predominantly moderate under the coefficient-adjusted classification thresholds. The mean RI value was 32.70, with a median of 32.17 and a range of 16.45–54.24. Among the 213 samples, 12 samples (5.6%) were classified as low risk, 200 (93.9%) as moderate risk, and one (0.5%) as considerable risk; no sample was classified as high risk. To visualize the spatial distribution of ecological risk, RI values were interpolated using the inverse distance weighting (IDW) in ArcGIS. As shown in Figure 5, the spatial distribution of RI exhibited pronounced heterogeneity along the river network, with elevated risk levels concentrated in scattered localized sections rather than forming extensive continuous high-risk zones. In sediments, relatively high RI values were concentrated primarily in the upstream reaches and in several transition sections between the middle and upper reaches, with additional elevated segments observed downstream, whereas relatively high RI values in cultivated soils occurred not only in upstream areas but also in certain downstream sections and tributary confluence zones. The middle reaches generally showed lower RI values, although localized increases were also observed near transition and confluence areas. Nevertheless, the occurrence of localized contamination hotspots and the distinct spatial patterns observed between cultivated soils and sediments highlight the influence of environmental heterogeneity on heavy-metal distribution and ecological risk.

### 3.2. Spatial Explanatory Power and Interaction Enhancement Based on GeoDetector

**Figure 6:**
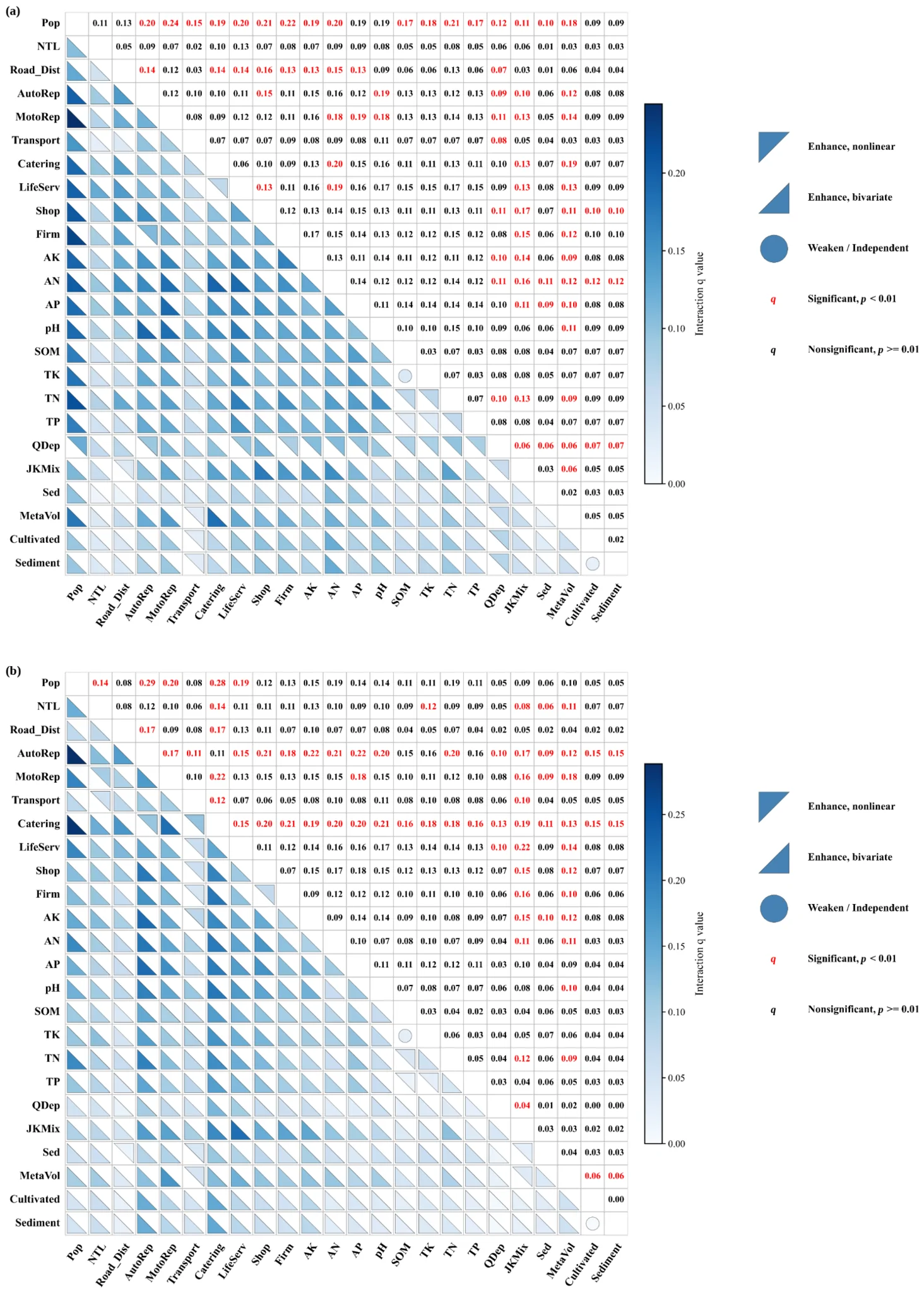

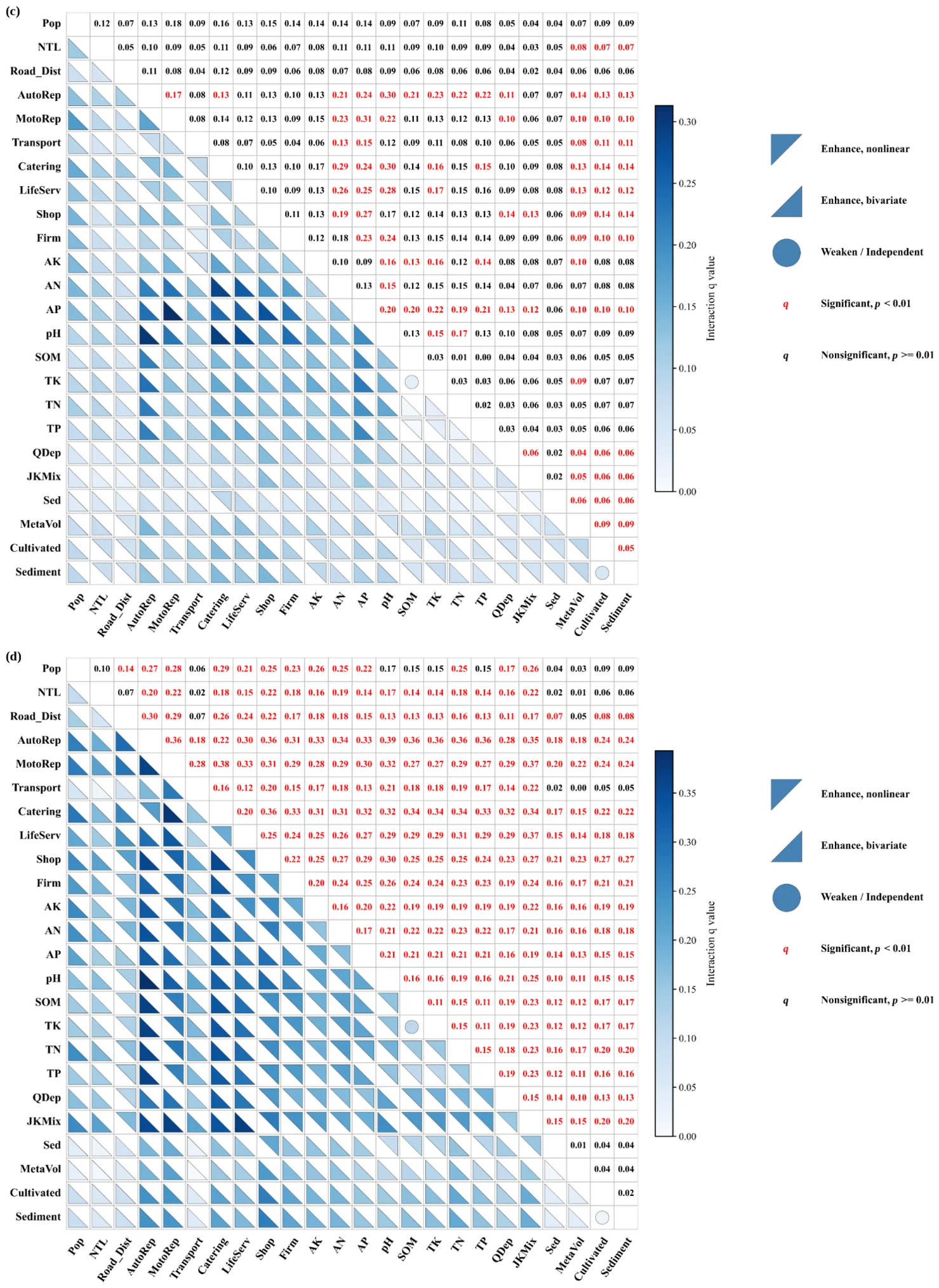

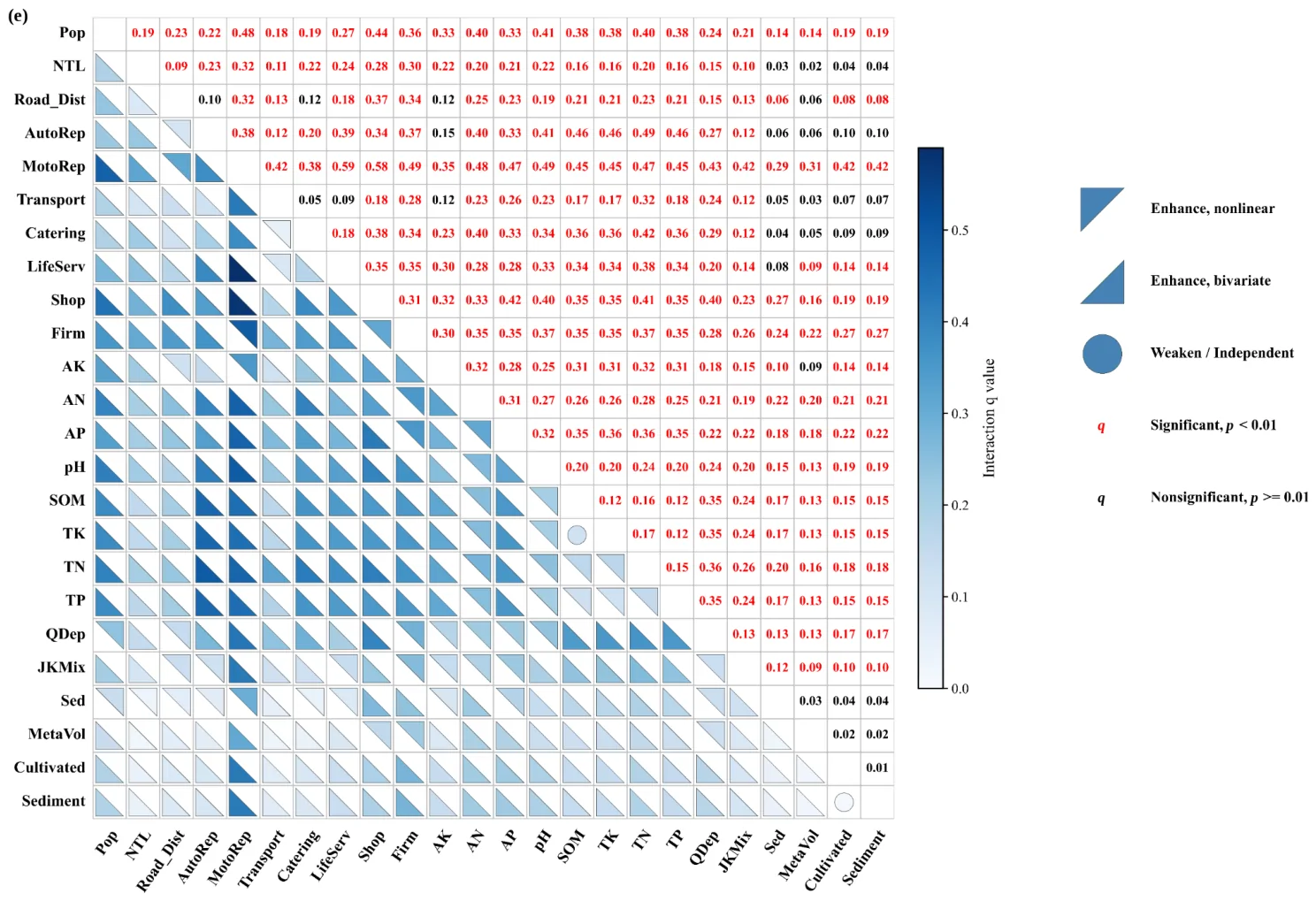
GeoDetector-based interaction effects of environmental factors on the spatial differentiation of heavy metals: (**a**) As; (**b**) Cu; (**c**) Pb; (**d**) Zn; and (**e**) Cr.

Figure 6 summarizes the interaction effects among environmental factors for the five heavy metals. Overall, interaction strengths varied substantially among metals, with relatively weak interactions for As, moderate interactions for Cu and Pb, pronounced enhancement for Zn, and the strongest interactive effects for Cr. Across all metals, factor combinations were predominantly characterized by bi-factor or nonlinear enhancement, indicating that heavy-metal spatial variability was better explained by factor combinations than by individual predictors.

For As, interaction effects were generally limited, with the highest explanatory power observed for population-related combinations, including Pop ∩ MotoRep (*q* = 0.24), Pop ∩ Firm (*q* = 0.22), and Pop ∩ Shop (*q* = 0.21). This pattern suggests that As spatial variability was primarily associated with the combined influence of population-related activities and environmental conditions. Cu exhibited stronger interactions between anthropogenic indicators and soil properties, with the highest *q* values observed for Pop ∩ AutoRep (*q* = 0.29) and Pop ∩ Catering (*q* = 0.28), followed by combinations involving available nutrients (e.g., AutoRep ∩ AK and AP ∩ Catering; *q* = 0.20–0.22). Pb showed a distinct interaction pattern dominated by the coupling of soil properties and localized human activities. The strongest interactions occurred for AP ∩ MotoRep (*q* = 0.31), pH ∩ AutoRep (0.30), pH ∩ Catering (0.30), and AN ∩ Catering (0.29), indicating that Pb differentiation was associated with the joint effects of soil conditions and activity-related factors rather than a single controlling variable. Zn exhibited extensive interaction enhancement, with high *q* values observed for pH ∩ AutoRep (0.39), MotoRep ∩ Catering (0.38), and AutoRep ∩ MotoRep and AutoRep ∩ Shop (both 0.36). Interactions involving geological indicators were also notable, particularly MotoRep ∩ JKMix and LifeServ ∩ JKMix (both 0.37), suggesting that parent-material conditions may contribute to Zn spatial variability. Cr showed the strongest interactive effects among the investigated metals. The highest *q* values were observed for MotoRep ∩ LifeServ (0.59) and MotoRep ∩ Shop (0.58), followed by MotoRep ∩ pH and TN ∩ AutoRep (both 0.49). Moreover, interactions between MotoRep and geological indicators (QDep and JKMix; *q* = 0.43 and 0.42, respectively) were relatively strong, highlighting the potential importance of both anthropogenic intensity and geological background in explaining Cr spatial heterogeneity.

Collectively, these findings demonstrate that heavy-metal spatial variability was characterized by metal-specific interaction patterns involving both natural background conditions and anthropogenic influences. The dominant interacting factors and their explanatory strengths differed among metals, reflecting distinct environmental associations during metal accumulation and redistribution. These GeoDetector results represent statistical explanatory relationships rather than direct causal effects or quantitative source apportionment.

### 3.3. Model Performance and Model-Derived Associations Based on XGBoost–SHAP

#### 3.3.1. Model Performance and Global Feature Importance

To evaluate the sensitivity of model performance to data partitioning strategies, pooled XGBoost models were trained using three training/testing ratios (8:2, 7:3, and 6:4) (Table 4). The optimal split was selected based on the highest evaluation-set *R*^2^ for each metal, resulting in 60:40 for As, 80:20 for Cu and Zn, and 70:30 for Pb and Cr. The selected models achieved *R*^2^ values of 0.385, 0.372, 0.355, 0.446, and 0.410 for As, Cu, Pb, Zn, and Cr, respectively. Corresponding RMSE values were 5.078, 7.370, 3.941, 12.912, and 88.582 mg kg^−1^. Given the dependence of RMSE on concentration scale, model performance comparisons were primarily based on *R*^2^. Overall, the models showed moderate explanatory capacity and were therefore interpreted as tools for identifying predictive associations and variable importance rather than for high-precision prediction or quantitative source attribution. Separate models constructed for cultivated soils and sediments showed negative evaluation-set *R*^2^ values and were not further interpreted. Additional details are provided in Supplementary Text S3.3.

**Figure 7:**
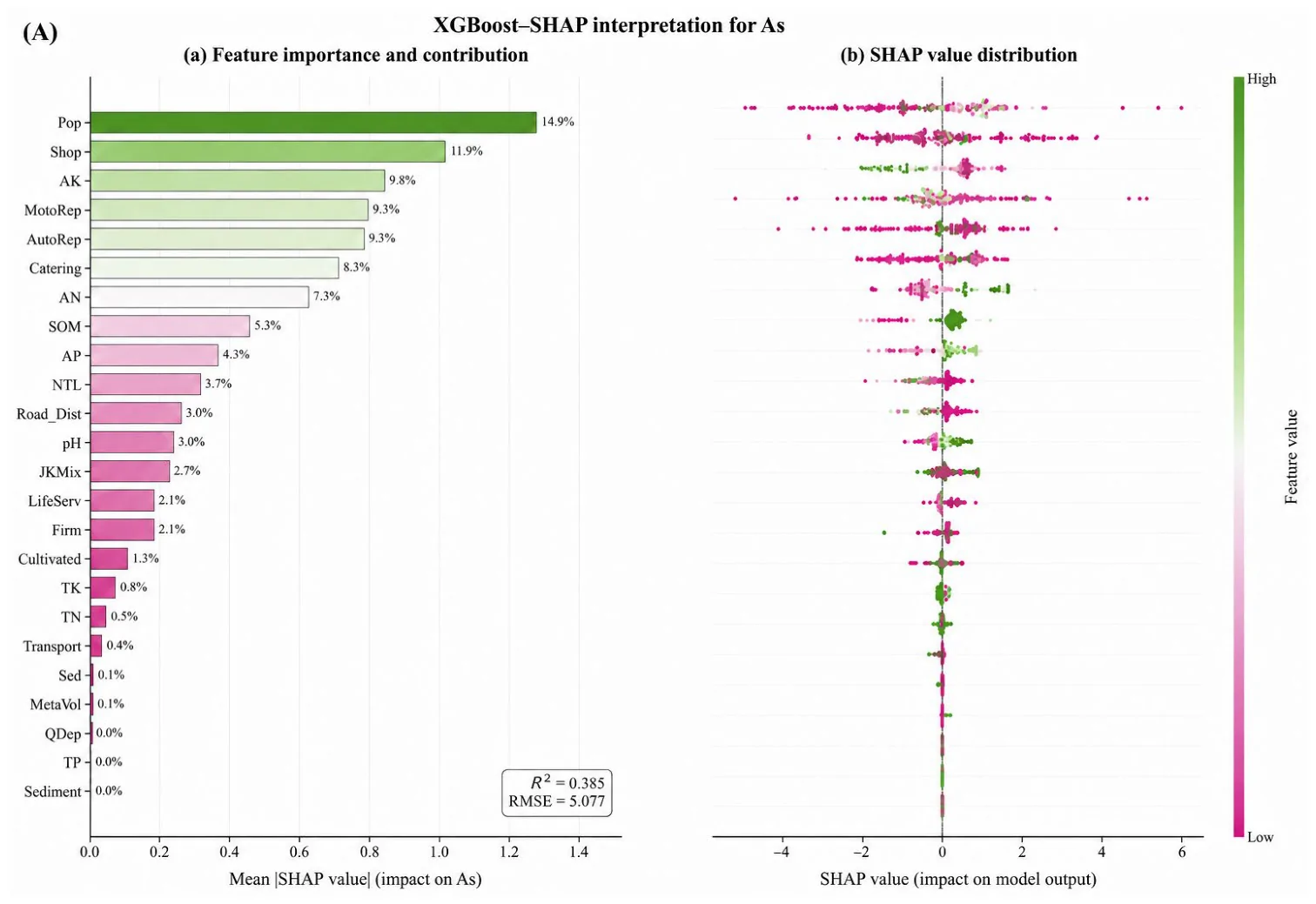

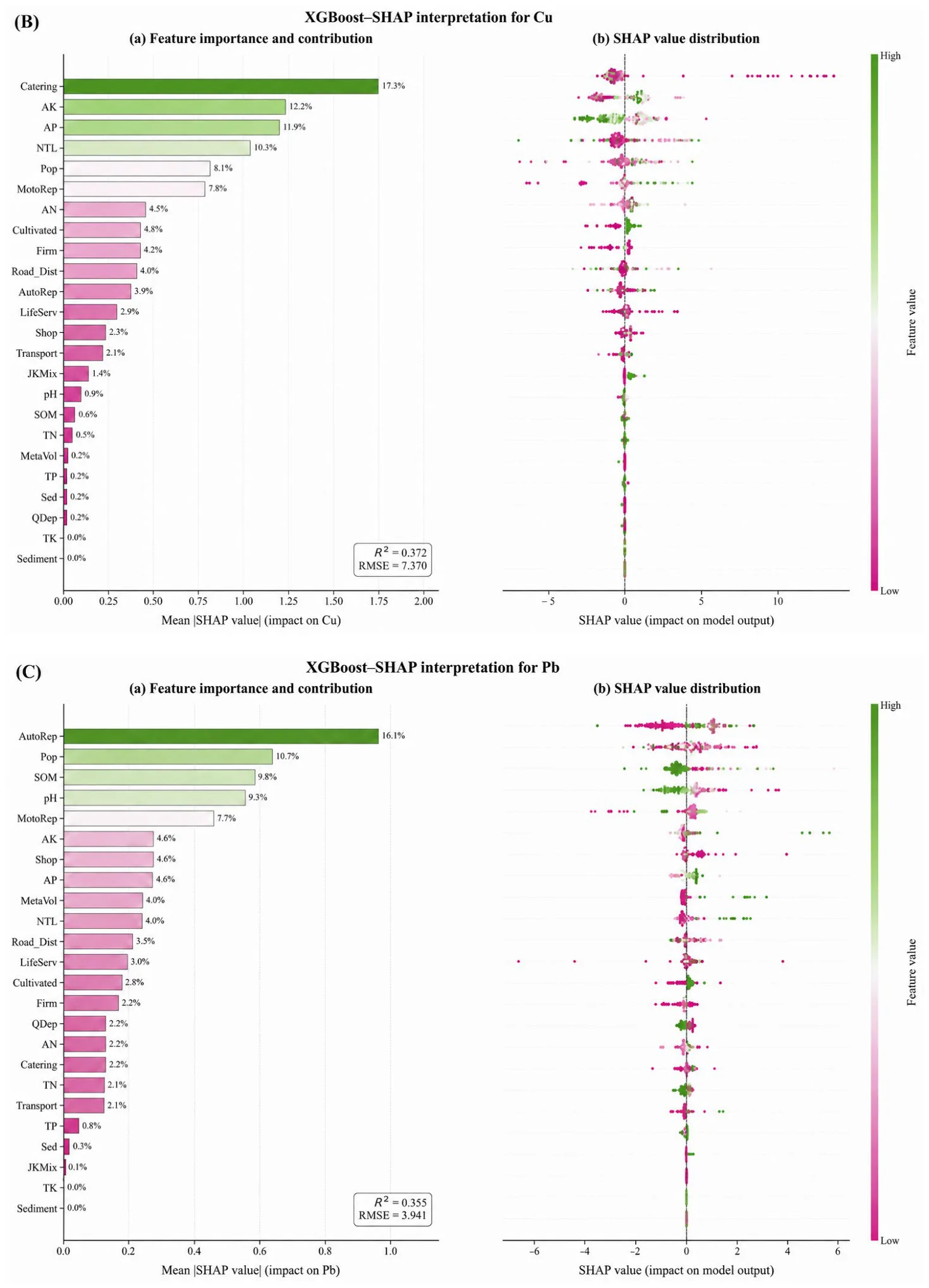

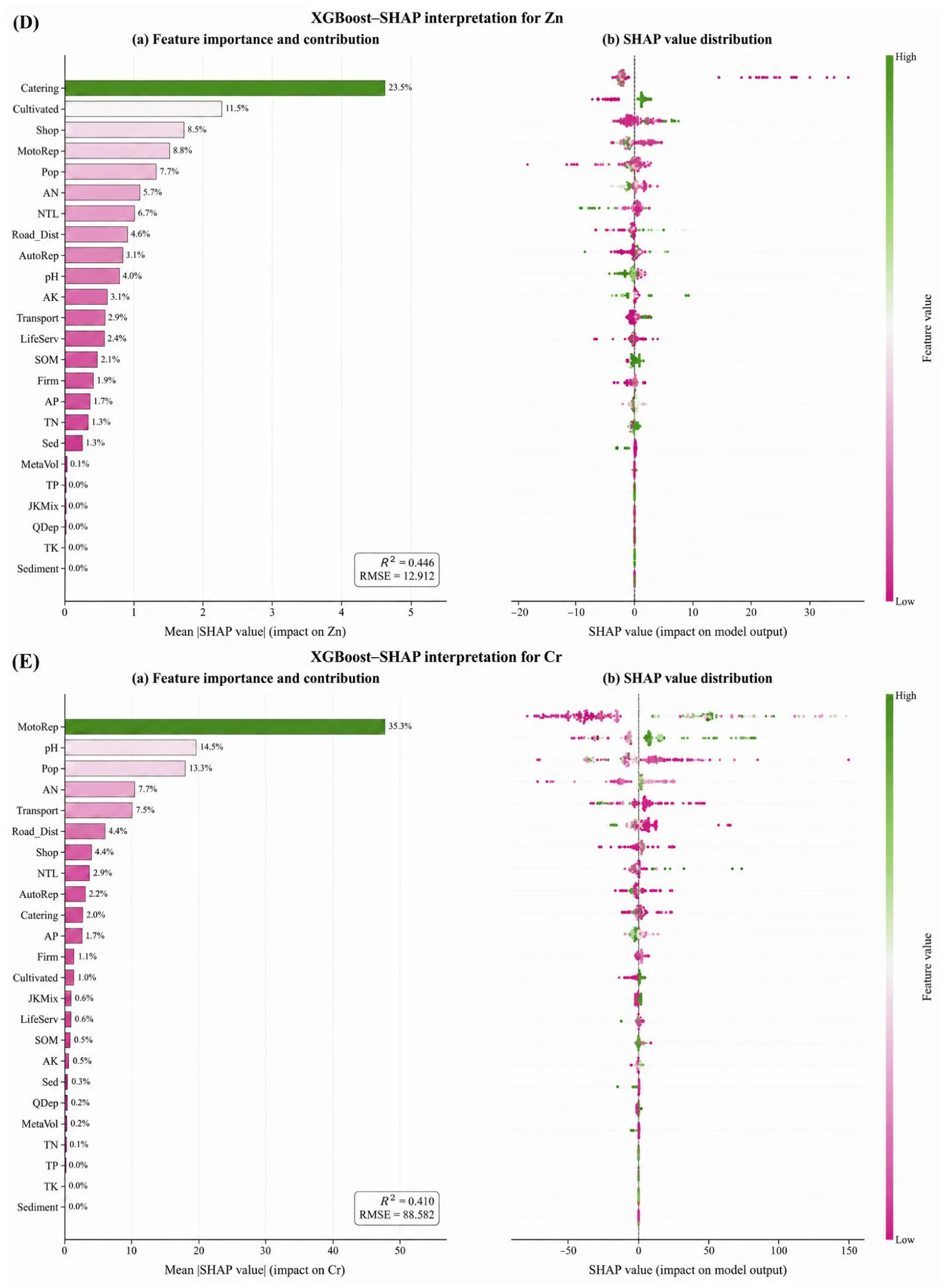
Evaluation-set performance and normalized global SHAP feature importance of pooled XGBoost models for (**A**) As, (**B**) Cu, (**C**) Pb, (**D**) Zn, and (**E**) Cr. Left: feature importance; right: SHAP value distribution.

As shown in Figure 7, the contribution profiles of environmental variables differed substantially among metals, indicating distinct predictive associations. The SHAP importance values represent normalized contributions of individual variables within each model. For As, predictive importance was relatively dispersed, with Pop (14.9%), Shop (11.9%), AK (9.8%), MotoRep (9.3%), AutoRep (9.2%), and Catering (8.3%) ranking among the most influential variables. This pattern suggests that As variability was primarily associated with population intensity, commercial activities, repair-related activities, and soil nutrient conditions [38].

For Cu, Catering (17.3%), AK (12.2%), AP (11.9%), NTL (10.3%), Pop (8.1%), and MotoRep (7.8%) showed the highest contributions, highlighting the combined relevance of localized human activities and soil fertility-related properties [39,40,41]. Pb exhibited a mixed contribution pattern, with AutoRep (16.1%) and Pop (10.7%) as the dominant variables, followed by SOM (9.8%), pH (9.3%), and MotoRep (7.7%). The comparable contributions of anthropogenic proxies and soil properties suggest that Pb variability was associated with both human activities and soil environmental conditions [42]. In addition, geological-background indicators collectively accounted for 6.6% of the SHAP importance, indicating a potential contribution of lithological characteristics to Pb prediction [12,13]. Zn showed the strongest model performance among the investigated metals. The major contributors included Catering (23.5%), medium type (Cultivated; 11.5%), Shop (8.8%), MotoRep (7.7%), Pop (6.7%), and AN (5.5%), suggesting important associations with human activity intensity, land-use characteristics, and soil nutrient conditions [43,44,45]. For Cr, predictive importance was highly concentrated, with MotoRep accounting for 35.3% of SHAP importance, followed by pH (14.5%), Pop (13.3%), AN (7.7%), and Transport (7.5%). Together, these variables contributed 78.3% of the total SHAP importance, indicating a dominant contribution structure. The high importance of MotoRep suggests a strong association between Cr variability and localized repair-related activities [45].

Overall, SHAP analysis revealed metal-specific predictive patterns involving anthropogenic activities, soil physicochemical conditions, and geological background. These importance rankings reflect model-based associations and should be interpreted within the predictive limitations of XGBoost models rather than as direct evidence of causal mechanisms or source contributions.

#### 3.3.2. SHAP-Derived Main Effects and Pairwise Interactions of Environmental Factors

**Figure 8:**
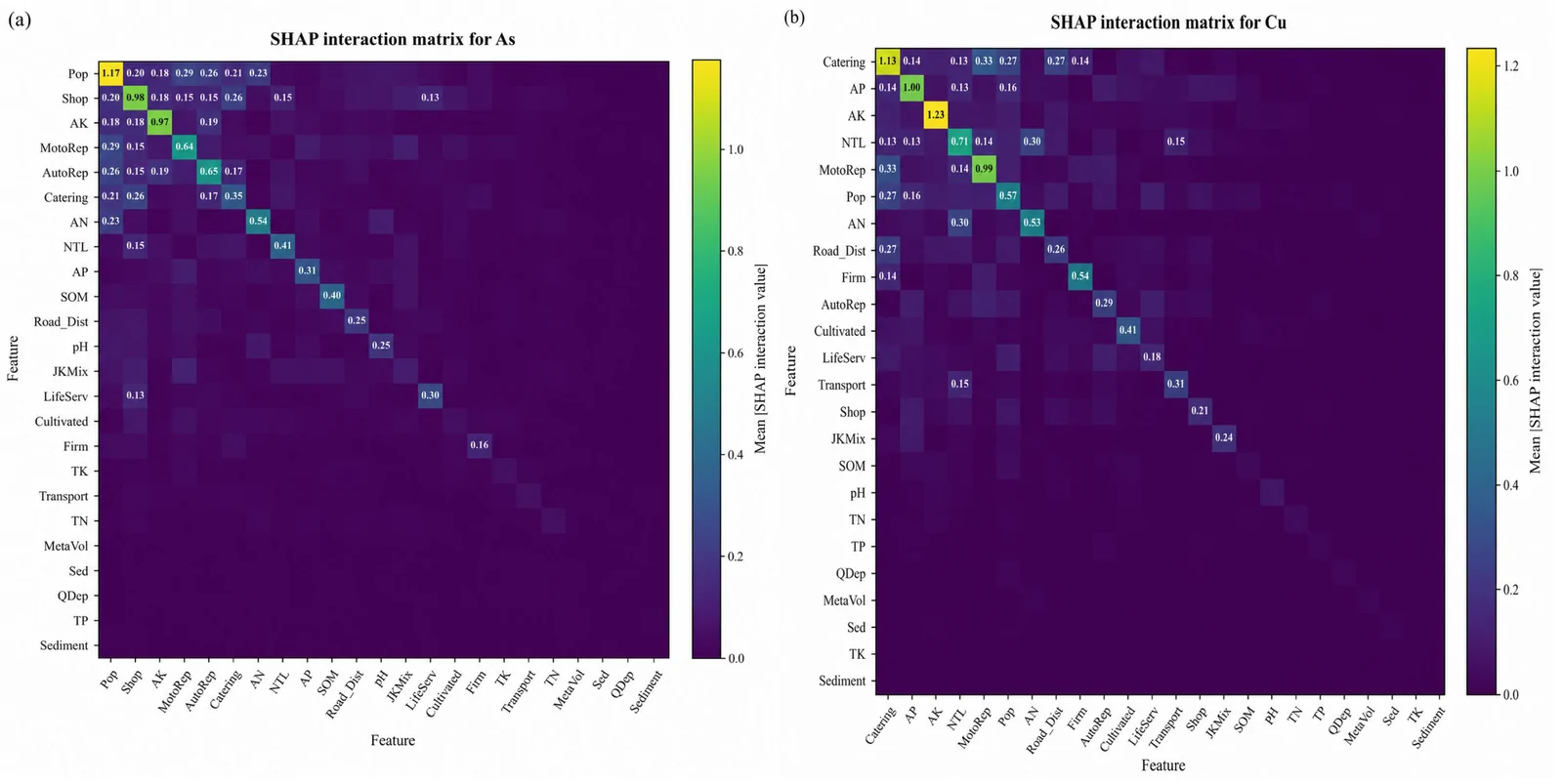

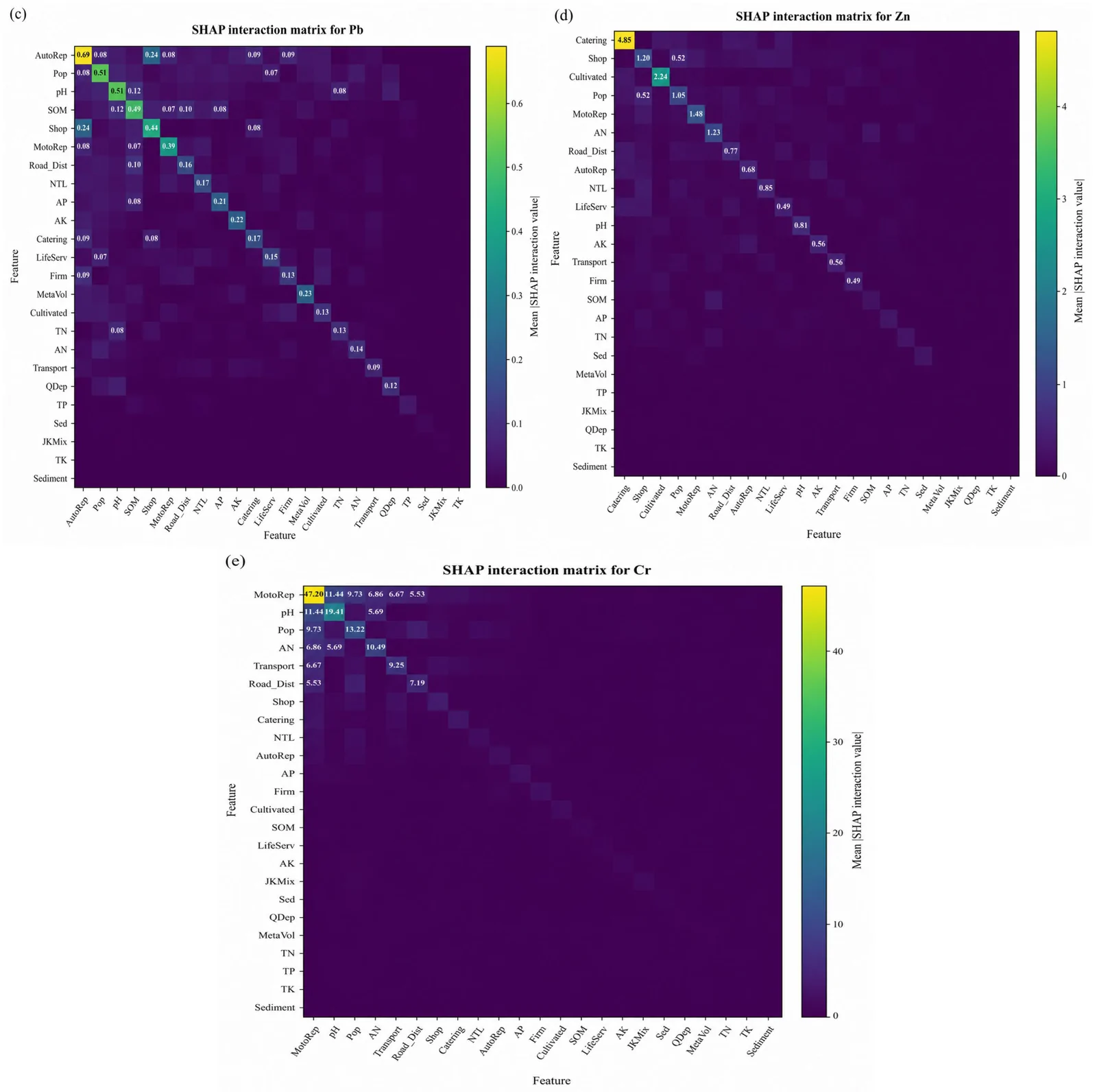
SHAP-derived main effects and pairwise interaction effects of environmental factors for (**a**) As; (**b**) Cu; (**c**) Pb; (**d**) Zn; and (**e**) Cr. Diagonal elements represent main effects, whereas off-diagonal elements represent pairwise interaction effects.

Figure 8 presents the SHAP-derived main effects and pairwise interaction contributions of environmental variables. Because SHAP interaction values are model-specific and scale-dependent, their magnitudes were interpreted within individual metal models rather than directly compared across metals.

For As, the largest main contributions were observed for Pop (1.17), Shop (0.98), AK (0.97), AutoRep (0.65), MotoRep (0.64), and AN (0.54). The dominant pairwise interactions included Pop ∩ MotoRep (0.29), Pop ∩ AutoRep (0.26), and Shop ∩ Catering (0.26), highlighting the combined contributions of population intensity, commercial activities, repair-related activities, and soil nutrient conditions to As predictions. For Cu, AK (1.23), Catering (1.13), AP (1.00), MotoRep (0.99), and NTL (0.71) showed the strongest main contributions. The most prominent interactions involved Catering ∩ MotoRep (0.33), NTL ∩ AN (0.30), and Catering-related combinations with Pop and Road_Dist (both 0.27), suggesting joint associations between urban activity intensity and soil nutrient characteristics. Pb exhibited a mixed contribution pattern, with AutoRep (0.69), Pop (0.51), pH (0.51), SOM (0.49), and Shop (0.44) showing relatively high main effects. The strongest interaction was AutoRep ∩ Shop (0.24), followed by pH ∩ SOM (0.12) and SOM ∩ Road_Dist (0.10). In addition, MetaVol showed a notable main contribution (0.23), indicating that geological background variables also contributed to Pb model predictions. For Zn, Catering showed the largest main contribution (4.85), followed by cultivated-land medium type (2.24), MotoRep (1.48), AN (1.23), Shop (1.20), and Pop (1.05). Compared with other metals, pairwise interactions were relatively weaker, with Shop ∩ Pop (0.52) representing the most prominent interaction. This pattern indicates that Zn predictions were primarily associated with human activity intensity, land-use characteristics, and soil nutrient conditions. Cr exhibited the most concentrated contribution structure, with MotoRep (47.20), pH (19.41), Pop (13.22), AN (10.49), and Transport (9.25) accounting for the major main effects. The strongest interactions were centered on MotoRep, particularly MotoRep ∩ pH (11.44), MotoRep ∩ Pop (9.73), MotoRep ∩ AN (6.86), and MotoRep ∩ Transport (6.67). These results suggest that Cr predictions were closely associated with repair-related activities and their interactions with soil chemical properties, population distribution, and transport-related factors [45].

Overall, SHAP-derived main effects and interaction patterns exhibited strong metal-specific characteristics. Anthropogenic indicators, soil properties, and geological background variables contributed differently across metals, highlighting distinct predictive association structures. The identified interactions, particularly those involving MotoRep in the Cr model, represent model-based associations and should be interpreted as exploratory relationships rather than direct evidence of causal mechanisms.

### 3.4. Local Contribution Patterns for Representative High-Concentration Samples

To further examine local contribution patterns in representative high-concentration samples, LIME was applied to selected cultivated-soil and sediment samples. Because LIME explains individual model predictions, the results were interpreted as exploratory local associations rather than direct source attribution. Contribution patterns varied among metals and environmental media.

**Figure 9:**
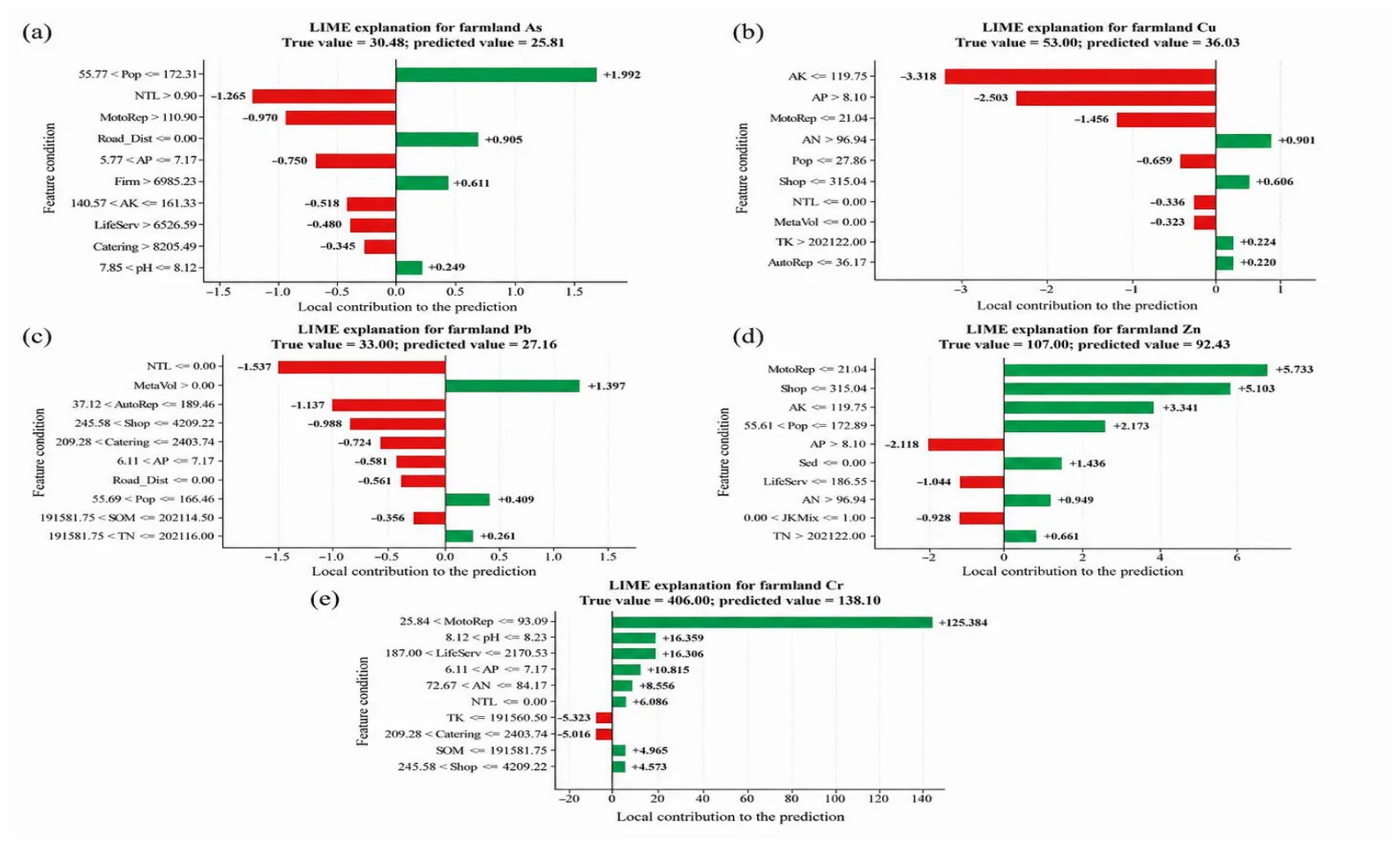
LIME explanations for representative high-concentration samples in cultivated soils: (**a**) As; (**b**) Cu; (**c**) Pb; (**d**) Zn; and (**e**) Cr.

Figure 9 shows distinct patterns for cultivated soils. For As, Pop and Road_Dist contributed positively, whereas NTL, MotoRep, AP, and AK contributed negatively. The predicted Cu concentration received negative contributions from AK, AP, and MotoRep, with positive contributions from AN and Shop. For Pb, MetaVol contributed positively, while NTL, AutoRep, Shop, Catering, and AP contributed negatively. The Zn prediction received positive contributions from MotoRep, Shop, AK, Pop, AN, and TN, whereas AP, LifeServ, and JKMix contributed negatively. For Cr, MotoRep made the largest positive contribution, followed by pH, LifeServ, AP, and AN.

**Figure 10:**
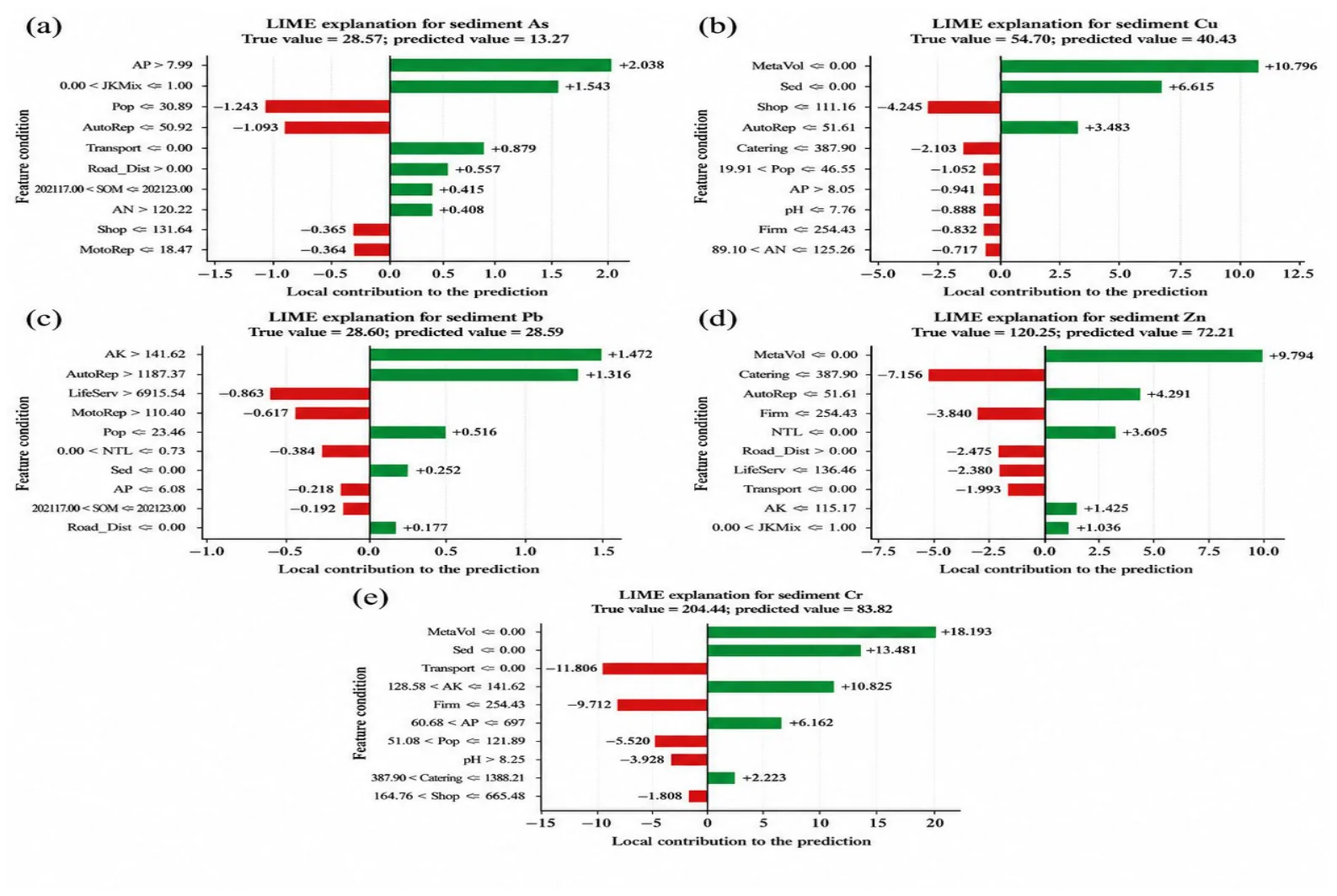
LIME explanations for representative high-concentration samples in sediments: (**a**) As; (**b**) Cu; (**c**) Pb; (**d**) Zn; and (**e**) Cr.

Figure 10 indicates different local patterns in sediments, with geological background showing a more prominent role than in cultivated soils. For As, AP and JKMix contributed positively, whereas Pop and AutoRep contributed negatively. Cu showed strong positive contributions from the geological-background indicators MetaVol and Sed, together with AutoRep. For Pb, AK and AutoRep were the main positive contributors, while LifeServ and MotoRep contributed negatively. Zn was positively associated with the geological-background indicators MetaVol and JKMix, as well as AutoRep and NTL. For Cr, MetaVol and Sed showed the largest positive contributions, followed by AK and AP. geological-background indicators showed relatively large local contributions to the selected sediment predictions for As, Cu, Zn, and Cr, indicating that geological-background variables were important contributors to these selected sediment predictions [13,46].

Overall, local contribution patterns differed across metals and environmental media. Cultivated-soil samples mainly reflected associations with anthropogenic activities and soil properties, whereas the model predictions for high-concentration sediment samples, particularly those of As, Cu, Zn, and Cr, were substantially associated with lithological and parent-material conditions.

### 3.5. Interpretation of Key Associated Factors and Pedological Implications

The identified statistical associations can be interpreted in relation to established environmental processes. Anthropogenic proxies, including population density, commercial activities, and repair-service indicators, represent the intensity of human disturbance associated with transportation, material abrasion, and localized waste inputs, rather than direct measurements of specific emission sources. In contrast, soil physicochemical properties, such as pH, SOM, and nutrient availability, regulate metal retention and mobility through processes including adsorption, complexation, precipitation, and competition for sorption sites. Therefore, these soil properties should be interpreted as regulatory factors influencing metal accumulation and redistribution rather than as pollution sources.

Across the integrated analytical approaches, anthropogenic indicators and soil physicochemical variables consistently emerged as important contributors, whereas geological-background variables showed stronger contributions in sediment samples, particularly in LIME-based local interpretations. GeoDetector further revealed strong interactions between Zn and Cr and lithological/parent-material indicators, while LIME identified geological variables as important contributors to high-concentration sediment samples of As, Cu, Zn, and Cr. These findings are consistent with previous studies emphasizing the influence of parent materials and geological inheritance on heavy-metal distributions in Tibetan Plateau environments [13,46]. The stronger geological signals in sediments likely reflect their role as sinks for weathering products, eroded soil particles, and upstream transported materials. In contrast, cultivated soils are additionally modified by agricultural practices, including fertilization, irrigation, tillage, and localized human activities, which may weaken the expression of natural geological controls. Overall, heavy-metal variability in the Nyangqu River Basin was associated with geological inheritance, pedological conditions, and anthropogenic indicators. These interpretations represent plausible explanations of statistical associations and should not be considered direct evidence of causal mechanisms.

### 3.6. Model Uncertainty and Interpretation Boundaries

The machine-learning framework employed in this study was designed primarily to identify potential drivers of heavy-metal spatial differentiation rather than to develop high-precision predictive models. Although XGBoost models explained part of the spatial variability in metal concentrations, predictive performance remained moderate, with evaluation-set *R*^2^ values ranging from 0.355 to 0.446 across the five metals.

These uncertainties likely arose from several sources. First, the relatively limited sample size and temporal variability among sampling locations may have restricted model generalization. Second, the integration of cultivated soils and sediments introduced additional environmental heterogeneity that may not have been fully represented by the available predictors. Third, some environmental covariates were derived from gridded datasets (1 km resolution), which characterize regional gradients rather than site-specific conditions. The mismatch between shared grid-based soil properties (151 samples within 49 grid cells), the gridded soil depth (0–4.5 cm), and field sampling depth (0–20 cm) may have further reduced the representation of fine-scale soil variability. In addition, anthropogenic variables, including POI density, population density, and nighttime light intensity, represent indirect indicators of human activity intensity rather than direct measurements of emission sources. Therefore, SHAP and LIME interpretations should be considered exploratory evidence of spatial associations and potential hotspot-related factors, rather than definitive attribution of pollution sources. Several association patterns recurred across GeoDetector, SHAP, and LIME analyses, indicating a degree of internal consistency across analytical approaches rather than independent validation.

Future studies should incorporate larger spatiotemporal datasets, direct emission-related indicators, refined land-use and anthropogenic activity proxies, and long-term monitoring of hydrological and sediment transport processes. Such improvements will help validate the potential factors identified here and advance mechanistic understanding of heavy-metal enrichment across interconnected environmental media.

## 4. Conclusions

Heavy metals in cultivated soils and sediments of the Nyangqu River Basin exhibited distinct element-specific enrichment patterns and spatially localized anomalies. Among the investigated metals, As showed persistent enrichment, whereas Cr displayed the greatest spatial variability and the most pronounced hotspot characteristics. The Nemerow Integrated Pollution Index indicated that slight pollution dominated at the sampling-site scale, while the coefficient-adjusted ecological risk assessment suggested that potential ecological risks were mainly within the moderate category. The spatial differentiation of heavy metals was associated with a combination of soil physico-chemical properties, geological background, and localized anthropogenic influences, with substantial metal-specific differences in the contribution of individual factors and their interactions. These findings highlight the heterogeneous and context-dependent nature of heavy-metal accumulation across environmental media. From a management perspective, monitoring efforts should prioritize localized high-concentration areas, particularly As- and Cr-enriched regions in cultivated soils and areas where elevated metal concentrations coincide with strong anthropogenic activity signals. However, potential pollution sources should be verified through field investigations and source-specific evidence before implementing targeted mitigation measures. Overall, this study demonstrates that relatively moderate regional ecological risk can coexist with localized high-concentration hotspots, emphasizing the importance of spatially explicit, element-specific monitoring strategies for risk identification and environmental management in alpine agricultural watersheds.

## Figures and Tables

**Table 1:** Data sources and spatial resolutions of environmental covariates.

Data Type	Data Source	Spatial Resolution
Soil physicochemical variables	Soil dataset from the National Tibetan Plateau Data Center (https://data.tpdc.ac.cn/home)	1 km
Roads	OpenStreetMap road vector data (https://www.openstreetmap.org)	Vector data
Population density	Gridded Population of the World, Version 4 (GPWv4): Population Density, Revision 11, NASA Socioeconomic Data and Applications Center (SEDAC)	1 km
Nighttime lights	NASA Black Marble Nighttime Lights product suite based on VIIRS Day/Night Band observations, VNP46A2, NASA LAADS DAAC	500 m
POI	Point-of-interest data from the AMap Open Platform (https://map.amap.com)	Point data
Geological background variables	SOTER-CN (https://www.isric.org/explore/soter/, accessed on 25 July 2026)	Vector data

**Table 2:** Software and computational environment used in this study.

Software or Package	Version	Application
PyCharm Community Edition	2023.3.7	Development, debugging, and execution of custom Python scripts
Python	3.9.13	Statistical analysis, GeoDetector implementation, machine-learning modeling, and data visualization
pandas	2.1.4	Data import, preprocessing, and tabular data management
NumPy	1.26.4	Numerical computation and array operations
SciPy	1.13.1	Statistical analysis and significance testing
Matplotlib	3.9.4	Visualization of GeoDetector and machine-learning analysis results
scikit-learn	1.5.2	Data splitting, cross-validation, hyperparameter optimization, and model evaluation
XGBoost	1.7.6	Construction of heavy-metal prediction models
SHAP	0.44.1	Global interpretation of feature contributions and nonlinear response patterns
LIME	0.2.0.1	Local interpretation of representative high-concentration samples
ArcGIS	10.8	Spatial data processing, inverse distance weighted interpolation, and map production
Origin	2024	Generation and formatting of boxplots and stacked percentage bar charts for heavy-metal concentrations and pollution-level distributions

**Table 3:** Heavy metal concentrations in farmland soils and sediments, 2019–2021 and 2024–2025.

Year	Medium	Element	Mean mg/kg	Maximum mg/kg	Minimum mg/kg	SD	CV(%)	Exceedance
2019	Cultivated soil (*n* = 17)	As	21.62	34.10	9.17	7.09	32.80	2
		Cu	40.27	65.00	29.50	8.75	21.73	0
		Pb	23.62	30.90	14.50	4.84	20.47	0
		Zn	90.28	116.0	67.70	13.51	14.97	0
		Cr	156.7	516.0	69.20	134.4	85.75	3
	Sediment (*n* = 7)	As	18.37	25.70	8.09	5.89	32.07	NA
		Cu	36.39	41.40	32.70	3.47	9.54	NA
		Pb	21.23	23.50	15.80	2.87	13.54	NA
		Zn	82.74	148.0	67.40	28.95	34.98	NA
		Cr	181.4	314.0	135.0	61.84	34.08	NA
2020	Cultivated soil (*n* = 31)	As	21.81	49.90	9.94	7.82	35.87	3
		Cu	38.26	56.60	21.60	7.73	20.20	0
		Pb	24.66	38.80	12.40	4.74	19.22	0
		Zn	93.15	146.0	70.60	17.25	18.52	0
		Cr	167.25	590.0	69.50	142.5	85.20	7
	Sediment (*n* = 11)	As	16.38	24.20	10.50	3.94	24.07	NA
		Cu	40.12	70.40	10.80	15.37	38.31	NA
		Pb	23.26	27.40	18.00	2.98	12.80	NA
		Zn	93.81	144.0	46.90	31.44	33.52	NA
		Cr	116.6	161.0	39.10	36.37	31.18	NA
2021	Cultivated soil (*n* = 51)	As	21.86	54.40	13.80	7.70	35.22	4
		Cu	35.75	71.70	20.60	9.25	25.89	0
		Pb	27.05	48.40	13.50	5.90	21.83	0
		Zn	88.05	128.0	68.40	13.10	14.87	0
		Cr	155.9	594.0	65.70	146.6	94.03	10
	Sediment (*n* = 9)	As	17.44	21.60	11.60	3.21	18.42	NA
		Cu	32.61	44.00	17.20	8.38	25.68	NA
		Pb	25.76	31.20	10.80	6.66	25.87	NA
		Zn	83.92	102.0	59.00	14.64	17.44	NA
		Cr	95.70	120.0	40.00	26.36	27.54	NA
2024	Cultivated soil (*n* = 38)	As	22.74	32.65	11.86	5.54	24.38	3
		Cu	40.34	52.28	28.45	6.29	15.59	0
		Pb	24.02	31.56	18.82	2.88	11.99	0
		Zn	82.01	114.5	61.55	11.57	14.10	0
		Cr	103.18	233.5	59.53	36.67	35.54	1
	Sediment (*n* = 29)	As	22.76	32.27	14.52	4.89	21.49	NA
		Cu	37.31	55.13	22.06	8.58	22.99	NA
		Pb	22.27	26.77	17.66	2.57	11.53	NA
		Zn	78.49	120.2	56.76	16.17	20.60	NA
		Cr	113.4	388.5	67.97	63.32	55.83	NA
2025	Cultivated soil (*n* = 12)	As	25.27	47.17	13.66	11.06	43.76	3
		Cu	35.96	44.10	23.91	5.01	13.94	0
		Pb	22.01	28.74	17.39	3.02	13.74	0
		Zn	69.09	91.39	50.33	11.14	16.13	0
		Cr	183.9	486.5	64.38	156.8	85.24	3
	Sediment (*n* = 8)	As	21.25	25.86	17.37	3.03	14.26	NA
		Cu	38.39	55.46	25.70	8.54	22.25	NA
		Pb	21.18	24.20	18.75	1.87	8.82	NA
		Zn	67.02	73.16	51.70	6.77	10.10	NA
		Cr	118.0	203.1	73.88	47.81	40.52	NA

Note: Exceedance sites were counted according to the risk-screening values for agricultural land in GB 15618–2018. This standard applies only to cultivated soils and not to sediments; therefore, sediment entries are denoted as NA.

**Table 4:** Sensitivity of pooled XGBoost model performance to different training-to-evaluation split ratios.

Element	*R* ^2^			RMSE		
	20%	30%	40%	20%	30%	40%
As	0.1343	0.3345	0.3850	5.0791	5.6052	5.0775
Cu	0.3716	0.2887	0.2334	7.3702	7.7186	7.8822
Pb	0.3153	0.3546	0.2432	3.4014	3.9410	4.0495
Zn	0.4457	0.3795	0.3940	12.9120	15.5750	14.3301
Cr	0.2585	0.4102	0.3722	102.7478	88.5819	96.0796

Note: The percentages indicate the proportions of samples assigned to the evaluation set; the split with the highest evaluation-set *R*^2^ for each metal was selected for subsequent SHAP analysis.

## Data Availability

The data presented in this study are available on reasonable request from the corresponding author.

## Supplementary Materials

The following supporting information can be downloaded at https://www.mdpi.com/article/10.3390/toxics14080697/s1, Table S1: Microwave digestion program for soil and sediment heavy-metal analysis; Table S2: Analytical accuracy for heavy-metal determination using the certified reference material GBW07548 (GSS-57); Table S3: Classification criteria for interaction effects identified by the GeoDetector interaction detector; Table S4: XGBoost hyperparameter tuning settings; Table S5: Representative high-concentration sample-selection criteria for LIME interpretation; Text S1: Calculation procedures and classification criteria for the single-factor pollution index, Nemerow Integrated Pollution Index, and potential ecological risk index; Text S2: Interaction classification criteria of GeoDetector; Text S3: Model implementation, performance, and interpretation strategy, including Text S3.1: GeoDetector, XGBoost–SHAP, and SHAP interaction settings; Text S3.2: Representative-sample selection for LIME interpretation; and Text S3.3: XGBoost performance and pooled-sample interpretation strategy.
