# Supplementary material for: Spatial Heterogeneity and Drivers of Heavy Metals in Soils and Sediments of the Nyangqu River Basin, Tibetan Plateau, China: Insights from GeoDetector and Explainable Machine Learning
Source: Toxics. 2026 Aug 6;14(8):697. doi: 10.3390/toxics14080697 (PMC13517251; doi:10.3390/toxics14080697)
Supplement: Supplementary file 1 [file toxics-14-00697-s001.zip › toxics-4423828-supplementary.pdf]

Spatial Heterogeneity and Drivers of Heavy Metals in Soils and Sediments of the Nyangqu River Basin, Tibetan Plateau, China: Insights from GeoDetector and Explainable Machine Learning

Jiale Chen [1,2], Geng Xu[2], Duo Bu[1,2], Xiaomei Cui[1,2], Junli Chen[2], Qiangying Zhang[1,2]* and Bo Fang[2,*]

Academic Editor(s): Nikiforos Alygizakis

Received: 23 June 2026
Revised: 2 August 2026
Accepted: 4 August 2026
Published: date

Copyright: © 2026 by the authors. Submitted for possible open access publication under the terms and

conditions of the Creative Commons Attribution (CC BY) license.

1 *Key Laboratory of Biodiversity and Environment on the Qinghai-Tibet Plateau, Ministry of Education, School of Ecology and Environment, Xizang University, Lhasa 850000, China;*
2 *Analysis and Testing Center, Xizang University, Lhasa 850000, China;*
* Correspondence: zhangqiangying@utibet.edu.cn (Q.Z.); fangb1994@gmail.com (B.F.); Tel.:+86-08916657596

Supplementary Materials

Table S1. Microwave digestion program for soil and sediment heavy-metal analysis.

Step	Ramp time (min)	Temperature program (°C)	Hold time (min)
1	7	Room temperature to 120	3
2	5	120–160	3
3	5	160–180	25

Table S2. Analytical accuracy for heavy-metal determination using the certified reference material GBW07548 (GSS-57)

Reference material	Element	Certified value (mg/kg)	Measured value (mg/kg)	Recovery (%)
GBW07548 (GSS-57)	As	15.2±0.5	16.5±0.7	108.6±4.6
	Cu	24±1	26.8±1.4	111.7±5.8
	Pb	24.9±1.3	23.4±1.2	94.0±4.8
	Zn	66±3	58.7±3.2	88.9±4.8
	Cr	74±5	78.9±3.6	106.6±4.9

Text S1. Calculation procedures and classification criteria for the single-factor pollution index, Nemerow Integrated Pollution Index, and potential ecological risk index

To evaluate heavy-metal accumulation and potential ecological risk in the study area, the single-factor pollution index (*Pi*), Nemerow Integrated Pollution Index (*Pn*), and comprehensive potential ecological risk index (RI) were calculated as follows:

$$P_i = \frac{C_i}{S_i} \quad \text{(S1)}$$

$$P_n = \sqrt{\frac{P_{\text{avg}}^2 + P_{\max}}{2}} \quad \text{(S2)}$$

$$RI = \sum_{i=1}^{m} \left(T_r^i \times \frac{C_i}{S_i} \right) \quad \text{(S3)}$$

where C_i is the measured concentration of heavy metal *i* in a sample, S_i is the corresponding background reference value, *P*avg is the mean of the single-factor pollution indices for all assessed metals at a sampling site, and *P*max is the maximum single-factor pollution index at that site. $T_r{}^i$ is the toxic-response coefficient of heavy metal *i*, and *m* is the number of heavy metals included in the assessment. The Tibetan soil background values used for As, Cu, Pb, Zn, and Cr were 16.2, 19.6, 27.6, 70.7, and 68 mg kg^{-1}, respectively.

The *Pn* classification criteria were: *Pn* ≤ 0.7, clean; 0.7 < *Pn* ≤ 1, warning level; 1 < *Pn*≤ 2,

slight pollution; $2 < Pn \leq 3$, moderate pollution; and $Pn > 3$, heavy pollution.

In the potential ecological risk assessment, the toxic-response coefficients used for As, Cu, Pb, Zn, and Cr were 10, 5, 5, 1, and 2, respectively, giving a summed toxic-response coefficient of 23. Because the original eight-substance Håkanson framework has a summed coefficient of 133, the conventional RI boundaries of 150, 300, and 600 were proportionally adjusted by a factor of 23/133. The resulting thresholds were: RI < 25.94, low risk; 25.94 ≤ RI < 51.88, moderate risk; 51.88 ≤ RI < 103.76, considerable risk; and RI ≥ 103.76, high risk.

The coefficient adjustment was applied only to the categorical interpretation of RI; the site-specific RI values were not altered. Application of the unmodified conventional threshold of RI < 150 would classify all 213 sampling sites as low risk. Therefore, the adjusted risk categories used in this study are specific to the present five-metal framework and are not directly comparable with classifications based on the conventional unmodified Håkanson thresholds.

Text S2. Interaction classification criteria of GeoDetector

The interaction detector classifies the interaction effects between two factors by comparing the q value after factor interaction, $q(X_1 \cap X_2)$, with the q values of the individual factors, $q(X_1)$ and $q(X_2)$, and their sum. The interaction types were defined as follows:

Table S3. Classification criteria for interaction effects identified by the GeoDetector interaction detector.

Interaction type	Criterion
Nonlinear weakening	$q(X_1 \cap X_2) < \min[q(X_1), q(X_2)]$
Single-factor nonlinear weakening	$\min[q(X_1), q(X_2)] < q(X_1 \cap X_2) < \max[q(X_1), q(X_2)]$
Bi-factor enhancement	$\max[q(X_1), q(X_2)] < q(X_1 \cap X_2) < q(X_1) + q(X_2)$
Independent	$q(X_1 \cap X_2) = q(X_1) + q(X_2)$
Nonlinear enhancement	$q(X_1 \cap X_2) > q(X_1) + q(X_2)$

Text S3. Model Implementation, Performance, and Interpretation Strategy

Text S3.1. GeoDetector, XGBoost-SHAP, and SHAP interaction settings

Continuous variables used in GeoDetector were discretized into five strata using quantile classification. When quantile classification was not feasible because of duplicate values or insufficient unique values, equal-interval classification was applied. Categorical variables, including medium type and binary geological-background indicators, were directly treated as strata.

For XGBoost modeling, three training-to-evaluation split ratios—80:20, 70:30, and 60:40—were evaluated using a fixed random seed of 42. Hyperparameters were optimized separately within the training subset for each split using GridSearchCV and three-fold K-fold cross-validation with shuffling. Negative mean squared error was used as the optimization criterion. For each metal, the split with the highest evaluation-set R^2 was selected for subsequent SHAP and LIME interpretation.

Global feature importance was calculated using mean absolute SHAP values and

normalized within each metal-specific fitted model. SHAP interaction values were summarized as mean absolute pairwise interaction effects. Because SHAP interaction magnitudes depend on the scale and fitted structure of each model, they were compared within, rather than across, metals. SHAP interaction values are model-derived quantities and are not equivalent to GeoDetector *q*-statistics.

Table S4. XGBoost hyperparameter tuning settings.

Item	Setting / Criterion
Train–test split	8:2,7:3 and 6:4
Random seed	42
Cross-validation	Three-fold K-fold cross-validation with shuffling
Hyperparameter optimization	GridSearchCV
Optimization criterion	Negative mean squared error
n_estimators	100, 200
max_depth	5, 8
learning_rate	0.05, 0.10

Text S3.2. Representative-sample selection for LIME interpretation

For LIME interpretation, representative high-concentration samples were selected using predefined rules rather than subjective selection. For cultivated soils, As and Cr candidates were selected according to the agricultural land risk-screening values in GB 15618–2018, whereas Cu, Pb, and Zn candidates were selected from the upper 10% of concentrations within cultivated soils because no exceedance was observed for these metals. For sediments, GB 15618–2018 was not applied because it is intended for agricultural soils rather than river sediments. Therefore, sediment candidates were selected from the upper 10% of concentrations within sediments and further screened using a local high-concentration criterion. The detailed LIME selection criteria and numbers of candidate samples are provided in Table S5. (The local_z value was calculated as the difference between the target sample concentration and the mean concentration of its five nearest neighboring samples, divided by the neighborhood standard deviation).

Table S5. Representative high-concentration sample-selection criteria for LIME interpretation.

**Medium**	**Metal**	**Selection criterion**	**Number of candidate samples**
Cultivated soil	As	As > 30 mg/kg	15
Cultivated soil	Cr	Cr > 200 mg/kg	24
Cultivated soil	Cu	≥ 90th percentile within cultivated soils	15
Cultivated soil	Pb	≥ 90th percentile within cultivated soils	15
Cultivated soil	Zn	≥ 90th percentile within cultivated soils	15
Sediment	As	≥ 90th percentile within sediments and local_z ≥ 0	4
Sediment	Cu	≥ 90th percentile within sediments and local_z ≥ 0	7

Medium	Metal	Selection criterion	Number of candidate samples
Sediment	Pb	≥ 90th percentile within sediments and local_z ≥ 0	8
Sediment	Zn	≥ 90th percentile within sediments and local_z ≥ 0	6
Sediment	Cr	≥ 90th percentile within sediments and local_z ≥ 0	7

Text S3.3. XGBoost performance and pooled-sample interpretation strategy

Before interpreting the SHAP results, the out-of-sample performance of the pooled XGBoost models was evaluated. To assess sensitivity to data partitioning, three training-to-evaluation split ratios—80:20, 70:30, and 60:40—were compared for each heavy metal. For each metal, the split yielding the highest evaluation-set R^2 was selected for subsequent interpretation. The selected splits were 60:40 for As, 80:20 for Cu and Zn, and 70:30 for Pb and Cr.

The selected models yielded evaluation-set R^2 values of 0.385, 0.372, 0.355, 0.446, and 0.410 for As, Cu, Pb, Zn, and Cr, respectively, with corresponding RMSE values of 5.078, 7.370, 3.941, 12.912, and 88.582 mg kg^{-1}. Overall, model performance was limited to moderate. The models were therefore used primarily for exploratory ranking of predictor importance and identification of potential model-derived associations, rather than for high-precision prediction or quantitative source apportionment.

SHAP importance values represent the relative contributions of predictors within each fitted model. They do not represent the proportions of total observed variance explained by individual variables, quantitative source contributions, or evidence of causal effects.

The global SHAP rankings were derived from pooled models incorporating both cultivated-soil and sediment samples. Separate cultivated-soil and sediment models produced negative evaluation-set R^2 values and were therefore not interpreted further. Inter-media differences were instead examined through concentration and ecological-risk comparisons, inclusion of the medium-type variable in the pooled models, and LIME-based local interpretation.
